# Delayed closed-loop neurostimulation for the treatment of pathological brain rhythms in mental disorders: a computational study

**DOI:** 10.3389/fnins.2023.1183670

**Published:** 2023-07-05

**Authors:** Thomas Wahl, Joséphine Riedinger, Michel Duprez, Axel Hutt

**Affiliations:** ^1^ICube, MLMS, MIMESIS Team, Inria Nancy - Grand Est, University of Strasbourg, Strasbourg, France; ^2^INSERM U1114, Neuropsychologie Cognitive et Physiopathologie de la Schizophrénie, Strasbourg, France

**Keywords:** neurostimulation, closed-loop, control, real-time, delay, EEG

## Abstract

Mental disorders are among the top most demanding challenges in world-wide health. A large number of mental disorders exhibit pathological rhythms, which serve as the disorders characteristic biomarkers. These rhythms are the targets for neurostimulation techniques. Open-loop neurostimulation employs stimulation protocols, which are rather independent of the patients health and brain state in the moment of treatment. Most alternative closed-loop stimulation protocols consider real-time brain activity observations but appear as adaptive open-loop protocols, where e.g., pre-defined stimulation sets in if observations fulfil pre-defined criteria. The present theoretical work proposes a fully-adaptive closed-loop neurostimulation setup, that tunes the brain activities power spectral density (PSD) according to a user-defined PSD. The utilized brain model is non-parametric and estimated from the observations via magnitude fitting in a pre-stimulus setup phase. Moreover, the algorithm takes into account possible conduction delays in the feedback connection between observation and stimulation electrode. All involved features are illustrated on pathological α- and γ-rhythms known from psychosis. To this end, we simulate numerically a linear neural population brain model and a non-linear cortico-thalamic feedback loop model recently derived to explain brain activity in psychosis.

## 1. Introduction

Electrical neurostimulation is an old human idea, and has been a well-established therapy for mental disorders for few decades. Caius Plinius during Antiquity and Scribonius Largus, who lived in the first century AD, proposed respectively contacts with the Electric ray (Torpedo Fish) for the treatment of post-partum pain and severe headaches. In the 19th century, electrical stimulation was commonly prescribed by neurologists for nervous disease (Edel and Caroli, 1987). Today, various electrical stimulation techniques exist to modulate neuronal systems and novel techniques for an optimal clinical treatment of a specific pathology gain more and more attention (Sun and Morrell, 2014; Chen et al., 2022). They could be used as an additional therapeutic lever or as an alternative to pharmacological medication, thus representing a hope for pharmaco-resistant forms of disease.

Brain oscillations result from coordinated electrical neuronal tissues activity within and between structures and networks. Implicated in various neural processes, such as perception, attention and cognition, their disruption yields pathological rhythms, which reflect abnormal activity of the implicated brain network, notably at the cellular and molecular level (Basar, 2013). These pathological rhythms serve as good biomarkers for neuropathologies. For instance, neurophysiological studies have revealed that a large number of mental disorders exhibit pathological rhythms, which do not occur in healthy patients (Schulman et al., 2011). Neurostimulation techniques have identified such pathological rhythms as good stimulation targets for the treatment of brain oscillatory disorders. Neurostimulation induces electric currents in neuronal tissue. Depending on the stimulation protocol, i.e. the temporal stimulation current shape, its duration and pause and the number of repetitions, neurostimulation can lead to neural plasticity effects or to pacemaker-like brain stimulation, respectively.

For example, Deep Brain Stimulation (DBS) is an invasive technique and proposed for patients suffering from severe pharmaco-resistant Parkinson's disease (PD) or obsessive-compulsive disorders. In PD patients aberrant hypersynchronicity and hyperactivity in the β-frequency band (12–30 Hz) of the basal ganglia-thalamocortical network can be addressed by the pharmacological medication (e.g. Levodopa) or DBS. The conventional DBS protocols focus on the subthalamic nucleus or globus pallidus stimulation continuously at a temporally constant frequency about 130 Hz. The suppression of the pathological beta oscillations was correlated with improving motor symptoms (Kühn et al., 2008). Recent techniques (Hosain et al., 2014; Fleming et al., 2020) propose to apply an adaptive closed-loop stimulation protocol based on observed intracranial brain activity. In addition to this intracranial neurostimulation technique, transcranial electrical stimulation (TES) and transcranial magnetic stimulation (TMS) are non-invasive neuromodulation approaches in which, respectively, a low electrical current and a magnetic field are applied to the cortical tissues. The TES current modalities include direct currents (tDCS), i.e. constant currents, alternating current (tACS), i.e. typically oscillatory currents, and random noise-shape currents (tRNS), which typically includes frequencies above the β-frequency band. It was shown that tDCS can improve cognitive performance in healthy subjects (Brunelin et al., 2012) and patients (Stagg et al., 2018) and it is applied as a therapeutic means to target brain network dysfunctions, such as Attention-Deficit/Hyperactivity Disorder (Nejati et al., 2020) and major depressive disorder (Bennabi and Haffen, 2018).

Although the neurostimulation techniques mentioned above may permit to alleviate mental disorder patients from symptoms, the success rate of these treatments is still limited (Nasr et al., 2022). This underperformance results from non-optimal choices of the stimulation protocol originating from the lack of understanding of the underlying neural response to stimulations and the non-patient specific stimulation protocol. In other words, typically the stimulation protocol (including size, duration, repetition cycle of the stimulation signal) is open-loop, i.e. pre-defined by the clinician based on heuristic criteria before the stimulation starts (Paulus, 2011). This non-optimal approach is inferior to so-called closed-loop techniques, which automatically adapt to the patients current brain/health state. Such an adaptive, or closed-loop, approach has been introduced for intracranial (Hartshorn and Jobst, 2018; Prosky et al., 2021; Stanslaski et al., 2022) and transcranial stimulation (Tervo et al., 2022) and has been shown to improve neurostimulation in major depression patients (Scangos et al., 2021), epilepsy (Haeusermann et al., 2023) and affective and anxiety disorders (Guerrero Moreno et al., 2021). These proposed closed-loop methods are adaptive in the sense that a pre-defined stimulation signal is applied when observed brain activity fulfills certain criteria, such as passing an amplitude or power threshold. While this adaptive approach improves existing open-loop methods, the pre-defined stimulation signal may still be non-optimally chosen. Recently proposed methods produce better results by using reference signal tracking control schemes such as proportional integral (PI) controller (Westover et al., 2015; Bolus et al., 2018; Su et al., 2019; Zhu et al., 2021), linear quadratic regulator (LQR) (Yang et al., 2018, 2019; Bolus et al., 2021) or model predictive control (MPC) (Fang and Yang, 2022, 2023) which uses an LQR in a MPC framework. However this form of control requires to pre-define a reference signal which is often non-trivial to provide in a patient specific manner. Furthermore, because of the stochastic nature of brain signals, forcing the resting state signal to follow a reference signal with its own independent noise creates an unnecessary constraint for the stimulation signal when we only want to regulate the power in given frequency bands.

We propose to estimate a stimulation signal on the basis of observed brain activity without the need to track a reference signal. The target stimulation signal is computed directly via a linear controller synthesized using a user-defined filter that encodes the desired frequency-domain modifications. We argue that it is the natural choice for a closed-loop optimization in the presence of pathological rhythms: typically the pathology is identified by an abnormal power in a certain frequency band and the closed-loop control aims to modify this power value in such a way that the final brain activity power spectral distribution resembles the distribution of a healthy subject. Examples are pathological too strong β-rhythm magnitudes in Parkinson's disease (Martin et al., 2018) and too weak α-rhythm (Howells et al., 2018) and too strong γ-rhythm in schizophrenia (Y et al., 2015). This approach implies the hypothesis that modifying the observed pathological brain rhythms of a patient to resemble brain rhythms of a healthy subject renders the patients brain state and improves the patients health situation. This assumption was motivated by the impressive improving impact of DBS in psychiatric disorders (Holtzheimer and Mayberg, 2011).

Technically, the proposed method aims to reshape the spectral distribution of observed data, such as electroencephalographic data (EEG). For illustration, we consider pathological brain rhythms observed in psychosis in the α- (Howells et al., 2018) and γ-band (Leicht et al., 2015). Our method relies on the extraction and the filtering in real-time of the brain resting state activity signal, using the EEG and an estimated brain response model. The underlying brain model is fully non-parametric and estimated from observed resting state EEG. Moreover, we consider the fact that the closed-loop feedback exhibits a certain conduction delay between measurement and stimulation. This conduction delay results from the transmission delay in the hardware and the numerical computation time of the stimulation signal. Very first estimates of this delay time are in the range of few tens of milliseconds (Private communication, Isope, 2020), i.e. in the range of EEG signal time scales. Consequently, the present feedback delay in real-world systems may affect the methods performance. Fang and Yang (2022) presents a method to increase the robustness of an adaptive closed-loop controller against delay by reducing the sensitivity of the closed-loop to high frequency disturbances. However, while this decreases generally the error in the closed-loop output, this also prevents to apply the control signal specifically to high frequency ranges like the γ-range. To our best knowledge, the present study is the first providing a method to effectively compensate the frequency-domain errors created by the feedback delay in closed-loop neurostimulation systems without sacrificing the controllability of high frequency ranges.

The remaining article is organized as follows: Section 2 presents the neurostimulation setup and the closed-loop circuit studied in the rest of this paper. Then, we propose a model-based controller design to apply desired modifications to the observed activity signal. Subsequently, we propose a model estimation method to extract the brain input response model needed for the controller design. Later, we address the problem of the closed-loop delay by designing an additional system to approximate the future values of the observations. Finally, we present two brain models, which illustrate and validate the proposed method. Then, Section 3 presents the simulation results of our circuits, including the accuracy of the model estimation step and the delay compensation. Lastly, in Section 4, we discuss the results of the method presented in the paper compared to the state of the art, mention limitations and pinpoint some perspectives and possible experimental tests.

## 2. Materials and methods

### 2.1. Simulated neurostimulation

We build a theoretical plant as a circuit containing a stimulation element and an observation element, both connected to the model brain system under study. In real practice, the stimulation element corresponds to the neurostimulation device, such as a TES system or a TMS coil. In contrast, the observation element may represent electro-/magneto-encephalographic electrodes (in the following called EEG) or electrodes observing Local Field Potential. We define the time-dependent functions *u*:ℝ → ℝ and *y*:ℝ → ℝ as the input stimulation current and the output EEG signal, respectively.

If no input current is applied, the output is a non-zero stochastic signal *y*_0_ corresponding to the measured resting state EEG activity and a non-zero neurostimulation current alters the output signal as a linear response. This alteration is caused by a change in the brain activity in response to the neurostimulation input and a direct measurement of the input current. The latter is undesirable as it is not correlated with brain dynamics but only with neurostimulation and measurement devices. In the following, we assume that observations include brain dynamics correlated output only while direct current measurements are filtered out. A method to remove the direct current measurement from the EEG signal is discussed in Section 4.

**Figure 1:**
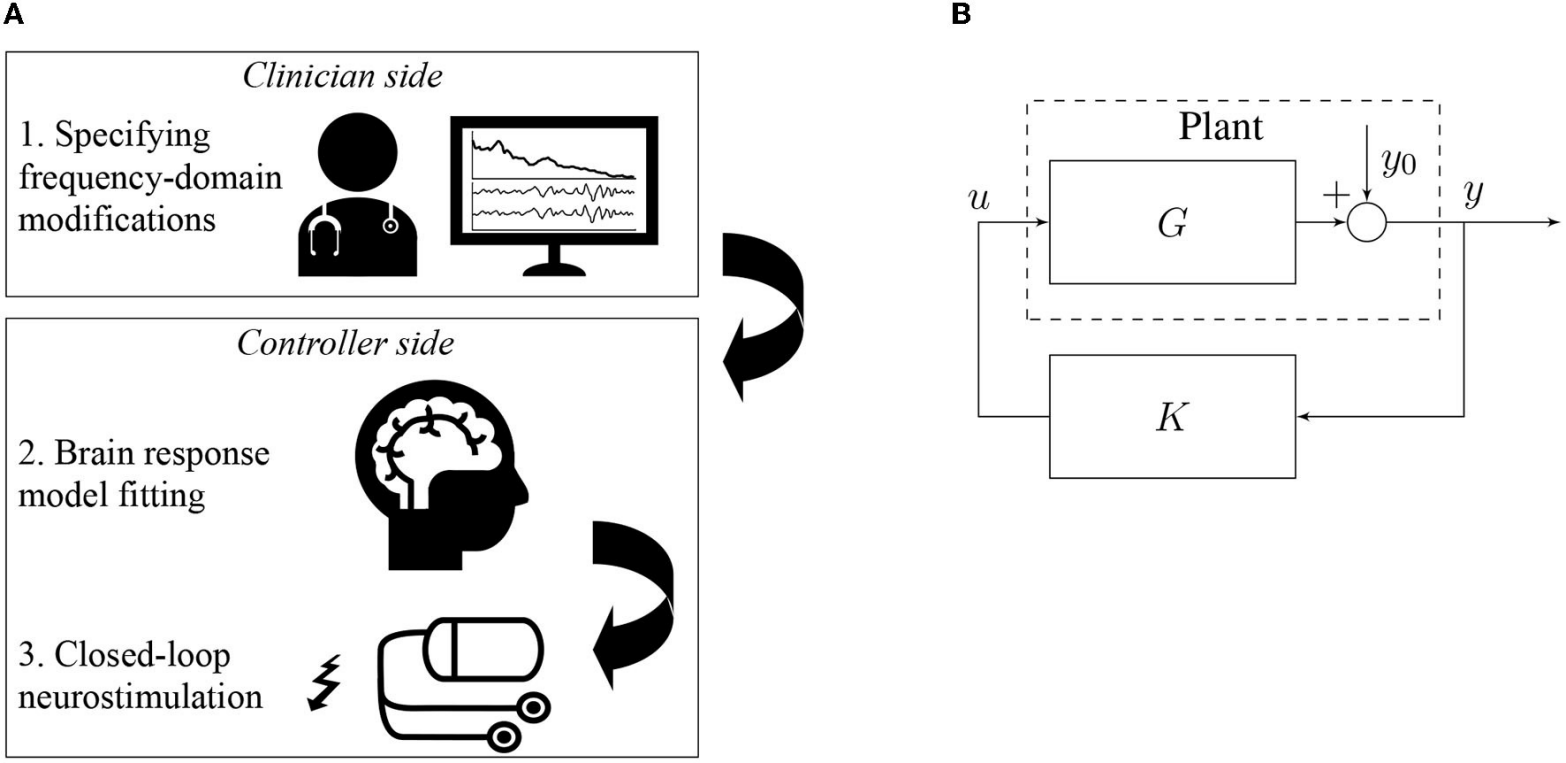
Closed-loop neurostimulation setup. **(A)** The different steps of our method in a possible experimental setup. First, the clinician specifies the desired frequency domain modifications to apply e.g. increase α-activity and decrease γ-activity. Second, the brain input response model is fitted to the measured EEG signal in order to match as closely as possible the current brain dynamics of the patient. Finally, the clinician's specifications and the fitted model are used to synthesize the closed-loop controller K that is directly used for closed-loop neurostimulation. **(B)** The controller directly produces the stimulation signal *u* in function of the measured total brain output *y* in real-time.

Then, we define the plant P as the system that takes *u* as its input and generates an output *y* which is equal to *y*_0_ when no input is applied. By modeling the dynamics of P, our goal is a neurostimulation signal *u* that causes predetermined changes in the spectral power amplitude of the output signal *y*. In our case, the goal is to increase the activity in the alpha band (8 − 12 Hz) and decrease the activity in the gamma band (25 − 55 Hz) motivated by aberrant power spectrum magnitudes in schizophrenia (Howells et al., 2018; Martin et al., 2018). A possible experimental setup involving our method is sketched in Figure 1A.

### 2.2. Linear time invariant model

We assume that the observed output response to a small neurostimulation input *u* is linear and time-invariant (LTI). This assumption is supported by multiple results across literature (Popivanov et al., 1996; Liu et al., 2010; Kim and Ching, 2016). Thus, there is an underlying LTI system G that produces an output *y*_*u*_ for any given input *u*. For this system, we can define a function *g*:ℝ → ℝ, which is the output produced by the plant input response system G in response to a unit impulse signal δ(*t*) where *t* ∈ ℝ is the time elapsed since the start of the signal. This function *g* is also called the unit impulse response of G and we have


$$\begin{array}{l} \begin{array}{l} y_{u}(t)=g(t)*u(t):=\int_{-\infty }^{+\infty }g(t^{\prime })u(t-t^{\prime })dt^{\prime }. \end{array} \end{array}$$

with time *t* and * denotes the convolution over time. It leads to the total plant output


(1) $$\begin{array}{l} y\left(t\right)=y_{0}\left(t\right)+y_{u}\left(t\right)=y_{0}\left(t\right)+g\left(t\right)*u\left(t\right). \end{array}$$

With this choice of model, the contribution of the neurostimulation response to the total output is purely additive, allowing us to focus the analysis on G, which represents the neurostimulation response part of the plant system. We also see that *y*_0_, the resting state activity, contains the stochastic part of the output, while *y*_*u*_ can be predicted for any known input signal *u* if we have a model for the system G. A method to estimate the plant input response model G is presented in Section 2.4.

### 2.3. Closed-loop control

In this section, we suppose that the function *g* is known. The estimation of *g* will be the aim of Section 2.4.

To close the loop, we generate the plant input signal *u* as the output of a linear controller K in response to the plant output *y*


$$\begin{array}{l} u\left(t\right)=k\left(t\right)*y\left(t\right), \end{array}$$

where *k*:ℝ → ℝ is the unit impulse response of the controller K. We can now rewrite Eq. (1) as


(2) $$\begin{array}{l} y\left(t\right)=y_{0}\left(t\right)+g\left(t\right)*k\left(t\right)*y\left(t\right). \end{array}$$

Here, we assume that no delay between observation and stimulation application is present. We will relax this condition in Section 2.5. To solve Eq. (2), we apply the Laplace transform defined for each time-dependent function *x*:ℝ → ℝ by


(3) $$\begin{array}{l} X(s)=L\{x(t)\}(s):=\int_{0^{-}}^{+\infty }x(t)e^{-st}dt, \end{array}$$

Thus, we define *Y*:ℂ → ℂ, *Y*_0_:ℂ → ℂ, *G*:ℂ → ℂ and *K*:ℂ → ℂ as the Laplace transforms of respectively *y*, *y*_0_, *g* and *k*, allowing us to write Eq. (2) as


$$\begin{array}{l} Y\left(s\right)=Y_{0}\left(s\right)+G\left(s\right)K\left(s\right)Y\left(s\right). \end{array}$$

Hence


(4) $$\begin{array}{l} Y\left(s\right)=\frac{1}{1-G\left(s\right)K\left(s\right)}Y_{0}\left(s\right). \end{array}$$

We now have an equation for the closed-loop output in function of the resting state activity. A block diagram of the closed-loop circuit is shown in Figure 1B.

Hence to design the frequency distribution of *y* we tune the frequency distribution of the transfer function *K* of the controller K.

#### 2.3.1. Controller synthesis

Our closed-loop setup aims to tune the observation power spectrum, or equivalently, the choice of *Y*(*s*) subjected to the resting state *Y*_0_(*s*). To this end, we define a linear filter H with transfer function *H*:ℂ → ℂ and


(5) $$\begin{array}{l} Y\left(s\right)=Y_{0}\left(s\right)+H\left(s\right)Y_{0}\left(s\right). \end{array}$$

Specifically, we intend to restore the physiological state of the brain, e.g., of a schizophrenic patient as our motivation, with an observed EEG presenting low alpha activity and high gamma activity. The chosen filter H is a weighted double bandpass filter with positive weight in the α-frequency band to increase α-power and negative weights in the γ-band to decrease the systems γ-activity. The filter's transfer function is defined as


$$\begin{array}{l} H\left(s\right)=c_{1}\frac{2\pi B_{1}s}{s^{2}+2\pi B_{1}s+{\left(2\pi f_{1}\right)}^{2}}+c_{2}\frac{2\pi B_{2}s}{s^{2}+2\pi B_{2}s+{\left(2\pi f_{2}\right)}^{2}}. \end{array}$$

The exact parameters of H are shown in Table 1.

**Table 1:** Parameter set of the filter H.

**Parameter**	**Description**	**Value**
*f*_1_	α-band natural frequency	10ms
*B*_1_	α-band width	4Hz
*c*_1_	α-band weight	1.0
*f*_2_	γ-band natural frequency	40ms
*B*_2_	γ-band width	30Hz
*c*_2_	γ-band weight	-0.5

The frequency parameters are chosen based on the alpha frequency range (8–12Hz) and the gamma frequency range (25–55 Hz) in an EEG. The weighting parameters *c*_1_ and *c*_2_, respectively positive and negative, corresponding to the choice to increase the alpha activity and decrease the gamma activity.

We can synthesize the closed-loop controller K, by combining equations (4) and (5) and solving for *K* as


(6) $$\begin{array}{l} \frac{1}{1-G(s)K(s)}Y_{0}(s)=Y_{0}(s)+H(s)Y_{0}(s)\;\\ \;K(s)=\frac{H(s)}{\left(1+H(s))G(s\right)}. \end{array}$$

Therefore, if we know the plant input response transfer function *G*, we can find that desired controller transfer function *K* by Eq. (6). Once the transfer function is obtained, we can use it to find a corresponding state-space representation (Hespanha, 2018) for time domain simulations. The state-space system's ordinary differential equations can then be implemented by any device that can measure the brain activity and produce a custom neurostimulation signal in real-time.

### 2.4. Model estimation

The design of our closed-loop controller requires estimating the plant input response system G, which in practice includes the brain dynamics, the neurostimulation device and the observation device. Our approach includes the estimation of G directly from observed brain activity, such as EEG of the patient. This ensure that the estimated plant model will be as close as possible to the real brain dynamics in the corresponding experimental conditions. To this end, we first need to find a way to measure the plant input response without also measuring the plant resting state activity. This is not trivial since the observed signal is the sum of the resting state activity and the stimulation response.

#### 2.4.1. Signal extraction

Let us consider an open-loop setup with an arbitrary input *u* applied to the plant, which generates the output described by Eq. (1). In this equation, we only know *u* and *y*, and want to estimate the impulse response *g*. The problem is that we cannot observe *y*_0_ only during the stimulation. Hence, based on previous data recordings, we need to find a way to predict the dynamics of *y*_0_ during the stimulation.

First, we provide the following standard definitions that are important in the subsequent discussion. For any time domain signal *x*:ℝ → ℝ, we denote the Fourier transform by


(7) $$\begin{array}{l} \hat{x}(f)=F\{x(t)\}(f):=\int_{-\infty }^{\infty }x(t)e^{-2\pi ift}dt. \end{array}$$

We define α_0_:ℝ → ℝ and α_*u*_:ℝ → ℝ such as α_0_(*t*) = *y*_0_(*t*)−ȳ_0_ and α_*u*_(*t*) = *y*_*u*_(*t*)−ȳ_*u*_ where ȳ, ȳ_0_ and ȳ_*u*_ are respectively the ensemble means of *y*, *y*_0_ and *y*_*u*_.

We assume that *y*_0_ is a wide-sense-stationary (WSS) random process, i.e. its mean and variance do not depend on time. According to the Wiener-Khinchin theorem (Khintchine, 1934; Gardiner, 2004), the autocorrelation function of a wide-sense-stationary random process has a spectral decomposition given by the power spectrum of that process


$$\begin{array}{l} S_{yy}\left(f\right)=|\hat{\alpha }\left(f\right){|}^{2}, \end{array}$$

where $\hat{\alpha }:\mathbb{R}\to \mathbb{C}$ is the Fourier transform of α(*t*) = *y*(*t*)−ȳ ∈ ℝ and $S_{yy}:\mathbb{R}\to {\mathbb{R}}^{+}$ is the spectral density of *y*.

Then, we can write Eq. (1) as


$$\begin{array}{l} ȳ+\alpha \left(t\right)={ȳ}_{0}+\alpha_{0}\left(t\right)+{ȳ}_{u}+\alpha_{u}\left(t\right), \end{array}$$

where ȳ = ȳ_0_+ȳ_*u*_. The equation then simplifies to


$$\begin{array}{l} \alpha \left(t\right)=\alpha_{0}\left(t\right)+\alpha_{u}\left(t\right). \end{array}$$

By application of the Fourier transform, we obtain


$$\begin{array}{l} \hat{\alpha }\left(f\right)={\hat{\alpha }}_{0}\left(f\right)+{\hat{\alpha }}_{u}\left(f\right) \end{array}$$

and


$$\begin{array}{l} |\hat{\alpha }\left(f\right){|}^{2}=|{\hat{\alpha }}_{0}\left(f\right){|}^{2}+|{\hat{\alpha }}_{u}\left(f\right){|}^{2}+2\text{Re}\left[{\hat{\alpha }}_{0}\left(f\right){\hat{\alpha }}_{u}{\left(f\right)}^{*}\right]. \end{array}$$

In the following, we compute the ensemble average of each term of this equation. Since α and α_*u*_ are two independent processes sampled at different times and $\langle {\hat{\alpha }}_{0}\rangle =\langle {\hat{\alpha }}_{u}\rangle =0$.

Hence


$$\begin{array}{l} \text{〈}2\text{Re}\left({\hat{\alpha }}_{0}\left(f\right){\hat{\alpha }}_{u}{\left(f\right)}^{*}\right)\text{〉}=2\text{Re}\left[\text{〈}{\hat{\alpha }}_{0}\left(f\right){\hat{\alpha }}_{u}{\left(f\right)}^{*}\text{〉}\right]=0. \end{array}$$

Here and in the following, 〈·〉 denotes the ensemble average. We point out that although Eq. (8) does hold when considering the ensemble average of the signals, fluctuations around 0 still remain in Eq. (8) for finite ensemble number of finite time signals.

We define the amplitude ratio (AR) as


(8) $$\begin{array}{l} \text{AR}=\langle \frac{|â\left(f\right){|}^{2}}{|{â}_{0}\left(f\right){|}^{2}}\rangle , \end{array}$$

which quantifies the gain of amplitude between the resting-state output and the stimulated output. The fluctuations mentioned above can also be reduced by increasing the input current which will lead to a higher AR and therefore, a higher contribution to the total signal of the â_*u*_ term (which is known) compared to the other terms.

Nevertheless, this yields


(9) $$\begin{array}{ll} \langle |{\hat{\alpha }}_{u}\left(f\right){|}^{2}\rangle & =\langle |\hat{\alpha }\left(f\right){|}^{2}\rangle -\langle |{\hat{\alpha }}_{0}\left(f\right){|}^{2}\rangle . \end{array}$$

Using Eq. (1), we can express ${\hat{\alpha }}_{u}$ in terms of the input impulse response *g* and the input *u*


(10) $$\begin{array}{l} {\hat{\alpha }}_{u}(f)=F\{y_{u}(t)-{\bar{y}}_{u}\}(f)\;\\ \;=F\{g(t)*[u(t)-\bar{u}]\}(f)\\ \;=\hat{g}(f)F\{u(t)-\bar{u}\}(f)\;. \end{array}$$

This equation permits to estimate the transfer function ĝ, see Section 3.

To express the transfer function ĝ in Laplace space, we use the fact that a unit impulse response function is non-zero only for positive time values *t*. Hence, based on equations (3) and (7), for *s* = 2π*if*, we can write the Laplace transform *G* as


$$\begin{array}{l} G(2\pi if)=\int_{0^{-}}^{+\infty }g(t)e^{-2\pi ift}dt=\int_{-\infty }^{+\infty }g(t)e^{-2\pi ift}dt=\hat{g}(f), \end{array}$$

where the unit impulse response function *g* directly relates the output *y* to the resting-state output *y*_0_ and the stimulation signal *u*, cf. Eq. (1).

We now need a method to generate a LTI system with a transfer function that matches the magnitude data computed with the formula. This is achieved by the magnitude vector fitting algorithm.

#### 2.4.2. Magnitude vector fitting

Our goal is now to find a transfer function *G* corresponding the magnitude data |ĝ(*f*)|^2^. For this purpose, we use a variant of the vector fitting algorithm design to work even with only the magnitude data. This method is called magnitude vector fitting (De Tommasi et al., 2010).

It allows to fit a passive LTI system to data by fitting the model transfer function. The system is synthesized such that the mean square error between the magnitude data sample and the transfer function evaluated at the same frequency points is minimized. De Tommasi et al. (2010) show that the transfer function of the fitted model reproduces both the magnitude and the phase shift of the original transfer function, although the fitting has been performed using sampled magnitude data only.

By minimizing the mean square error, the algorithm ensures that the transfer function of the fitted model accurately matches the original model as represented by the reconstructed gain data. Furthermore, to assess the accuracy of the reconstruction, we also compare the fitted model to the transfer function of the linearized brain model used for the simulation. This allows to double-check the validity of the reconstructed magnitude and also to verify if the reconstructed phase fits the phase of the original model as closely as possible (cf. Figures 3C, D).

We define the root-mean-square error (RMSE) as


(11) $$\begin{array}{l} \text{RMSE}=\sqrt{\langle |\frac{\tilde{G}\left(2\pi if\right)-G\left(2\pi if\right)}{G\left(2\pi if\right)}{|}^{2}\rangle }, \end{array}$$

where $\tilde{G}$ is the fitted model's transfer function, *G* is the original model's transfer function and *f* ∈ ℝ^+^ are the frequency points used for the fitting. This allows to quantify the accuracy of the fitting step.

### 2.5. Delay compensation

Realistic feedback loops exhibit conduction delays between the moment of observation and feedback stimulation. Reasons for such delays are finite conduction speeds in cables, electronic switches, interfaces and delays caused by the controller device to compute numerically adapted stimuli. In systems with large time scales, such as controlled mechanical devices on the centimeter or larger scale, such delays may be negligible. Conversely biological systems such as the brain evolve on a millisecond scale and conduction delays may play an important role. Preliminary estimation of input and output devices of desktop computers have revealed an approximate delay of ~10ms. By virtue of such delays, it is important to take them into account in the closed-loop between the moment of observation and stimulation.

The different sources of delay can be represented as plant input and output delays. Since the controller K is LTI, the input and output delays can be concatenated into one single plant input delay. Hence, in our setup, we model the delay as an input delay τ in the system G, modifying *y*(*t*) = *g*(*t*)*u(*t*) in Eq. (1) to *y*(*t*) = *g*(*t*)*u(*t*−τ). The Smith predictor (Smith, 1959; Morari and Zafiriou, 1989) is a known method to compensate such delay times. However, in the present problem, this approach allows controlling a limited frequency band only (Figures 7A, B). Consequently, it was necessary to invent another method. Since the plant input *u* is generated by the controller K, we modify the controller to compensate the delay. To this end, the new controller K is chosen to estimate the future value of *u* instead of the present value. The new proposed method to apply this controller modification is presented in the Results Section 3.2.

### 2.6. Comparison to the state of the art

Our method is tested against two main control schemes commonly used in adaptive closed-loop neurostimulation. These control schemes are closed-loop control with a PI controller (Westover et al., 2015; Bolus et al., 2018; Su et al., 2019; Zhu et al., 2021) and Linear Quadratic Gaussian (LQG) control (Yang et al., 2018, 2019; Bolus et al., 2021) which refers to the combination of a Linear Quadratic Estimator (LQE) (or Kalman filter) with a Linear Quadratic Regulator (LQR) (Åström, 2012). For both these methods, the tracked reference signal is generated from pre-recorded pathological resting state activities to which we apply a filter restoring the target α- and γ-activities. In order to prevent closed-loop destabilization and regulate high frequency disturbances, a Smith predictor (Smith, 1959) is used for the PI and LQG controller to compensate the delay, while we use our own delay compensation method for our controller.

### 2.7. Brain models

Our closed-loop control method works for any LTI brain model. Furthermore, we want to show that it also produces good results on non-linear brain models, for which the neurostimulation input response behaves closely to an LTI system, when the input is sufficiently small. To this end, we present two models used to test our method. The first one is a linear neural population model of cortical activity, and the second one is a non-linear cortico-thalamic neural population model with cortico-thalamic delay.

#### 2.7.1. Linear brain model

We describe neural population activity with a noise-driven linear model (Hutt, 2013). The model is composed of two pairs of interacting excitatory and inhibitory populations. Here we have $V_{e,i}^{\left(1,2\right)}:\mathbb{R}\to \mathbb{R}$, representing the mean activity of the associated population, where $V_{e}^{\left(1,2\right)}$ and $V_{i}^{\left(1,2\right)}$ correspond respectively to excitatory and inhibitory populations. Each population is driven by noise ξ_1, 2_:ℝ → ℝ and the external input *u*:ℝ → ℝ, according to the following differential equations:


(12) $$\begin{array}{l} \left\{ \begin{array}{l} \tau_{e,1}\frac{dV_{e}{}^{\left(1\right)}(t)}{dt}\text{ = }(-1+N_{11})V_{e}^{\left(1\right)}(t)-N_{11}V_{i}^{\left(1\right)}(t)+b_{1}u(t)+\xi_{1}(t),\\ \tau_{i,1}\frac{dV_{i}{}^{\left(1\right)}(t)}{dt}\;=N_{21}V_{e}^{\left(1\right)}(t)+(-1-N_{21})V_{i}^{\left(1\right)}(t)+b_{2}u(t),\\ \tau_{e,2}\frac{dV_{e}{}^{\left(2\right)}(t)}{dt}=(-1+N_{12})V_{e}^{\left(2\right)}(t)-N_{12}V_{i}^{\left(2\right)}(t)+b_{3}u(t)+\xi_{2}(t),\\ \tau_{i,2}\frac{dV_{i}{}^{\left(2\right)}(t)}{dt}=N_{22}V_{e}^{\left(2\right)}(t)+(-1-N_{22})V_{i}^{\left(2\right)}(t)+b_{4}u(t), \end{array}\right. \end{array}$$

where the noise ξ_1, 2_ is uncorrelated Gaussian distributed with zero mean and variance $\kappa_{1,2}^{2}=10^{-7}$, and the stimulation *u* is weighted by the coupling constants *b*_*i*_>0 of the corresponding population. In addition, τ_(*e, i*), (1, 2)_ are the synaptic time constants of the populations, and constants *N*_*ij*_>0 are interaction gains of the respective population. Table 2 provides the parameters employed in subsequent simulations.

**Table 2:** Parameter set of model (12).

**Parameter**	**Description**	**Value**
τ_*e*, 1, 2_	exc. synaptic time constant	5ms
τ_*i*, 1, 2_	inhib. synaptic time constant	20ms
*N*_11_	first exc. linear coefficient	1.15
*N*_21_	first inhib. linear coefficient	0.63
*N*_12_	second exc. linear coefficient	2.52
*N*_22_	second inhib. linear coefficient	6.6
*N*	number of neurons	1000
$\kappa_{1,2}^{2}$	noises variances (pathological)	10^−4^/*N*
${\left(\kappa_{1}^{\prime }\right)}^{2}$	first noise variance (healthy)	3.6·10^−4^/*N*
${\left(\kappa_{2}^{\prime }\right)}^{2}$	second noise variance (healthy)	2.5·10^−5^/*N*
*b*_1, 2_	input coupling constants	0.18
*b*_3, 4_	input coupling constants	0.14

The choice of parameter is partially based on the paper in which it was developed (see Hutt, 2013). The difference between the healthy and pathological state is modeled by changes in the amplitude of the finite size fluctuations of each population.

**Figure 2:**
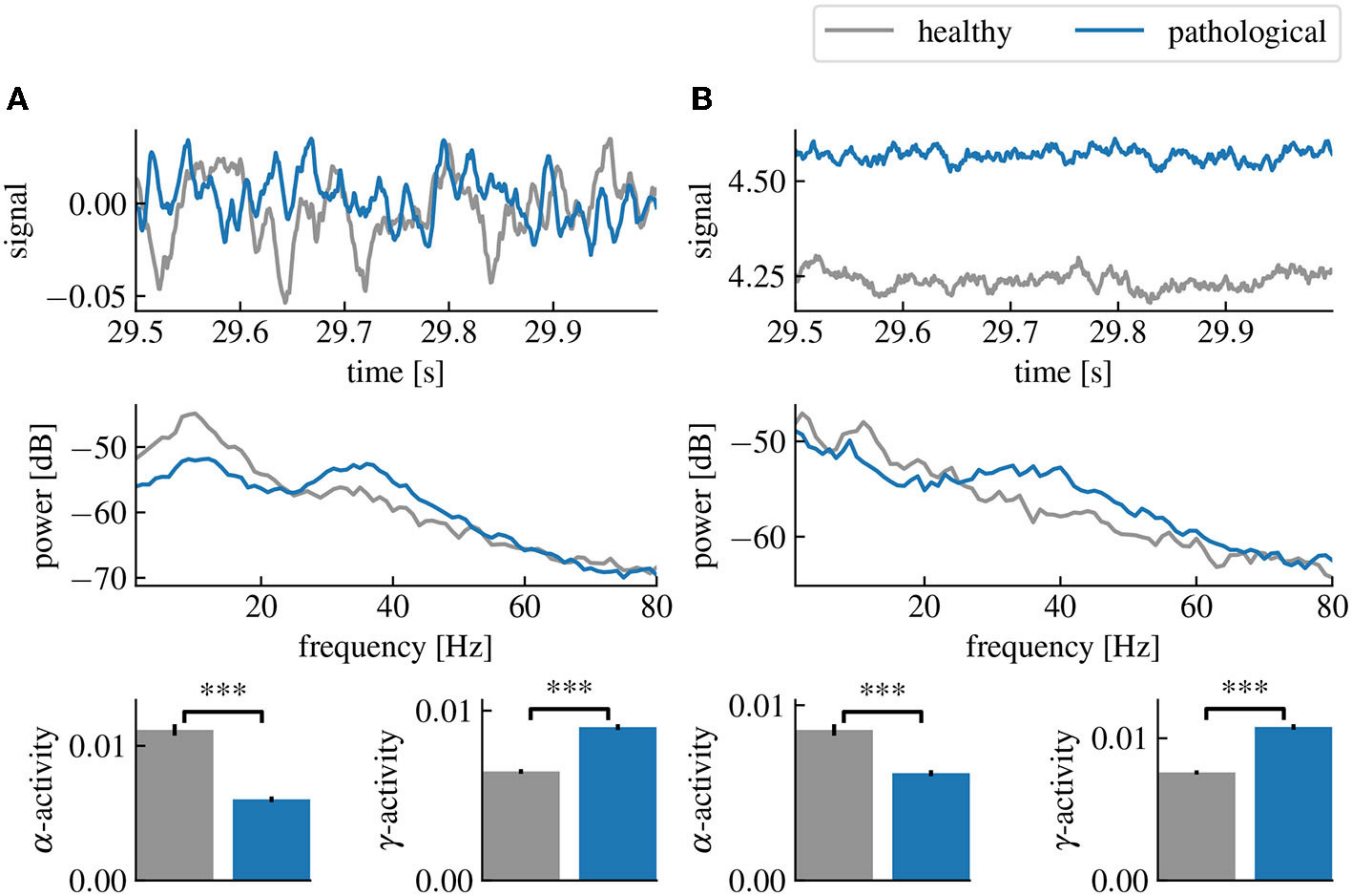
Healthy and pathological resting state activity of the linear and cortico-thalamic brain models. The pathological state is characterized by a decreased α-activity and an increased γ-activity. **(A)** Comparison of the healthy and pathological state of the linear brain model. The first row shows the last 500 ms of the simulated time series. The second row shows the power spectral densities estimated from the time series and the last row shows the estimated α- and γ-activities computed from the spectral densities and averaged over 50 simulations. **(B)** Same as **(A)** but using the cortico-thalamic brain model. “***” corresponds to a *p*-value less than 0.0005 using Welch's *t*-test.

The observed output


$$\begin{array}{l} y\left(t\right)=V_{e}^{\left(1\right)}\left(t\right)-V_{i}^{\left(1\right)}\left(t\right)+V_{e}^{\left(2\right)}\left(t\right)-V_{i}^{\left(2\right)}\left(t\right) \end{array}$$

is a sum of the effective field potential $V_{e}^{\left(j\right)}-V_{i}^{\left(j\right)}$ of both populations *j* = 1, 2, (cf. Figure 2A).

The simulation of the linear brain model in time domain is done using the library control of python. The numerical integration is computed thanks to matrix exponential (Van Loan, 1978), with a simulation sampling time of 1ms. The resting state activity of the linear brain model in shown in Figure 2A.

#### 2.7.2. Cortico-thalamic brain model

A different model considers the cortico-thalamic feedback circuit (Riedinger and Hutt, 2022). It describes the cortex layers I-III and the cortico-thalamic loop between cortical layers IV-VI, the thalamic relay cell population and the reticular structure. The cortical layer I-III exhibits mean activity of excitatory cells *v* and inhibitory cells *w*. Similarly, layer IV-VIs exhibits the mean activity *V*_*e*_ and *V*_*i*_ and thalamic relay cell populations the mean activity *V*_*th,e*_ and *V*_*th,i*_. Moreover, the reticular structure has the mean activity *V*_*ret*_. The fibers between the cortex and thalamus and the cortex and reticular structure exhibit a finite conduction delay τ (Hashemi et al., 2015; Riedinger and Hutt, 2022). The 7-dimensional dynamical system of the brain state $\text{x}=\left(v,w,V_{e},V_{i},V_{th,e},V_{th,i},V_{ret}\right)\in {\mathbb{R}}^{7}$ obeys


(13) $$\begin{array}{l} \left\{ \begin{array}{l} \overset{\cdot }{\text{x}}(t)\;=\text{F}(\text{x}(t),\text{x}(t-\tau ))+\xi (t)+\text{B}u(t),\\ y(t)\;=\text{Cx}(t), \end{array}\right. \end{array}$$

where the superscript *t* denotes transposition, **F** ∈ ℝ^7^ is a nonlinear vector function, **B** ∈ ℝ^7 × 1^ is the input coupling matrix and C ∈ ℝ^1 × 7^ is the observation matrix. We mention that $\text{B}={\left(b_{1},b_{2},b_{3},b_{4},0,0,0\right)}^{t},b_{i}>0$, i.e. only the cortical layers are stimulated with weights *b*_*i*_. The observation *y* captures the activity of the cortical excitatory populations (Nunez and Srinivasan, 2006; Riedinger and Hutt, 2022) with C = (*c*_1_, 0, *c*_3_, 0, 0, 0, 0), *c*_*i*_>0. For more details, please see the Supplementary material.

The time domain simulations of the cortico-thalamic model is done by numerical integration using the fourth-order Runge-Kutta method implemented by the scipy library in python with a maximum simulation time step of 1 ms. The resting state activity of the cortico-thalamic brain model is shown in Figure 2B.

### 2.8. Measuring brain activity

The general activity in EEG measurement is measured by first estimating the power spectral density of the signal using frequency bins of 1 Hz and then summing all the frequency bins up to the Nyquist frequency. In practice, the sum will be mostly determined by the activity in low frequencies and more precisely, near the α- and γ-activity peaks. To measure the results of our method, we define the α- and γ-activities as the sum of 1Hz frequency bins only in their respective frequency bands, i.e., respectively 8–12 Hz and 25–55 Hz. Meanwhile, the total activity of the neurostimulation signal, that we call the mean amplitude of *u* is computed from 0 Hz to the Nyquist frequency.

## 3. Results

The present work addresses two major problems in closed-loop control: the correct model choice of the systems dynamics and the present conduction delay. The subsequent sections propose solutions for both problems and illustrate them in some detail by applying them to the linear brain activity model from Section 2.7.1. The final section demonstrates the closed feedback loop for the cortico-thalamic brain model from Section 2.7.2.

### 3.1. Model estimation

Equations (9) and 10 permit to express the magnitude of ĝ(*f*) in terms of the spectral densities of observable signals


(14) $$\begin{array}{l} |\hat{g}(f){|}^{2}|F|u(t)-\bar{u}\}(f){|}^{2}=\;|\hat{\alpha }(f){|}^{2}-|{\hat{\alpha }}_{0}(f){|}^{2}\;\\ \;|\hat{g}(f){|}^{2}S_{uu}(f)=S_{yy}(f)-S_{y_{0}y_{0}}(f)\\ \;|\hat{g}(f){|}^{2}=\frac{S_{yy}(f)-S_{y_{0}y_{0}}(f)}{S_{uu}(f)}. \end{array}$$

**Figure 3:**
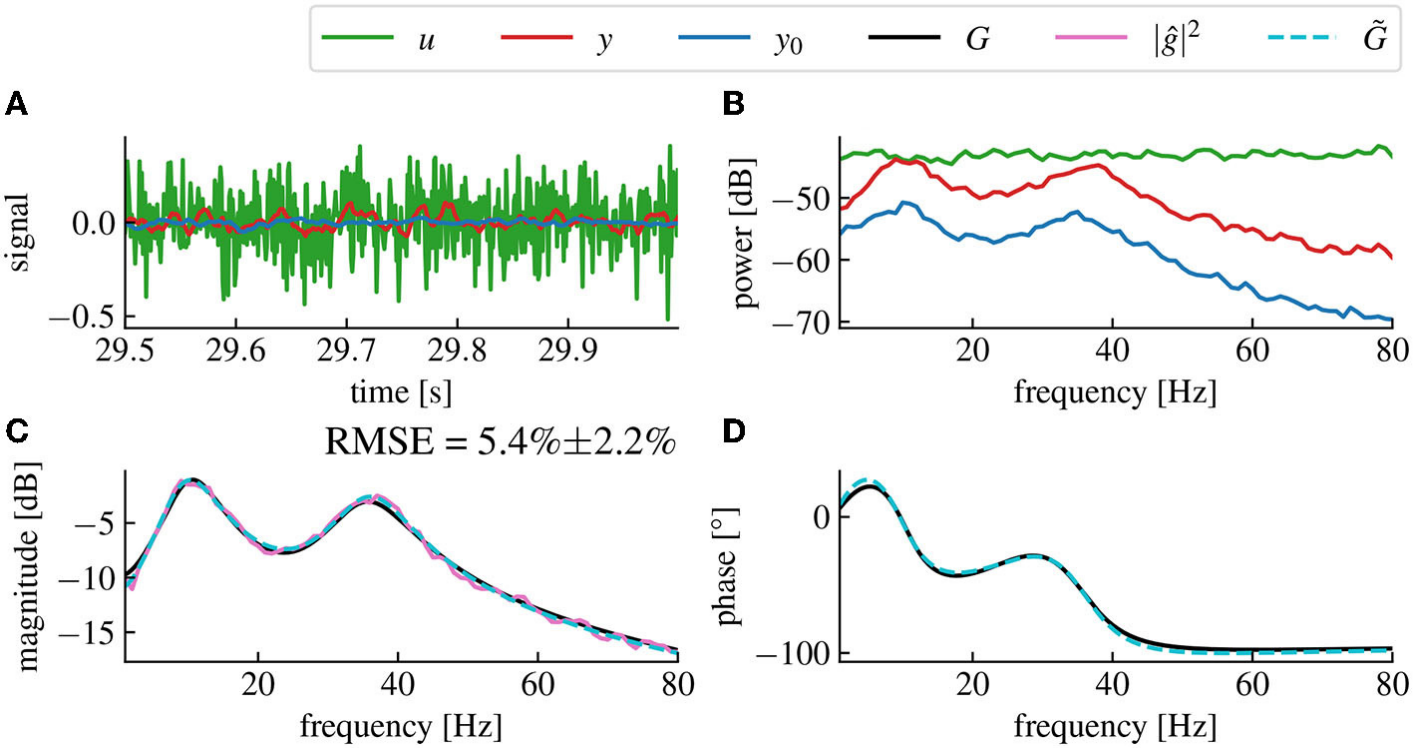
The magnitude vector fitting algorithm successfully reconstructs the transfer function *G* from magnitude-only data. **(A)** Time series of the resting state activity (blue), the input signal (green) and the stimulation response (red). **(B)** Spectral densities of the simulated input signal (green), the resting state activity (blue) and the stimulation response (red). The input signal is a white noise with chosen standard deviation 0.005. **(C)** Reconstructed gain |ĝ| of the plant input response. The fitted model (dashed cyan) accurately matches the original model (black) with a root mean square error (RMSE) of 5.4%±2.2% (Confidence Interval (CI) 95%). The RMSE represents the error percentage between the fitted model's transfer function and the original model's transfer function averaged over 50 trials. The pink curve is the raw data used for fitting, computed from the spectral density data in **(A)** using Eq. (14). **(D)** Reconstructed phase of the plant input response ĝ.

The spectral density functions *S*_*y*_0_*y*_0__ and *S*_*yy*_ may be estimated numerically from output data before and during a stimulation with a known chosen stimulation function *u*. The estimation may be performed by applying conventional methods, such as the Welch method (Welch, 1967). These estimations provide the magnitude of the transfer function |ĝ| by utilizing Eq. (14). In detail, at first, we considered the linear model (12) and injected a white noise current into the plant gaining the system's response signal together with the resting state activity, (cf. Figure 3A). The subsequent estimation of *S*_*yy*_(*f*), *S*_*y*_0_*y*_0__(*f*) and *S*_*uu*_(*f*) (Figure 3B) from the data permitted to compute the brain input response model ĝ(*f*) by Eq. (14). We observe a very good accordance of the original model response function and its estimation in magnitude (Figure 3C) and phase (Figure 3D).

#### 3.1.1. Robustness

**Figure 4:**
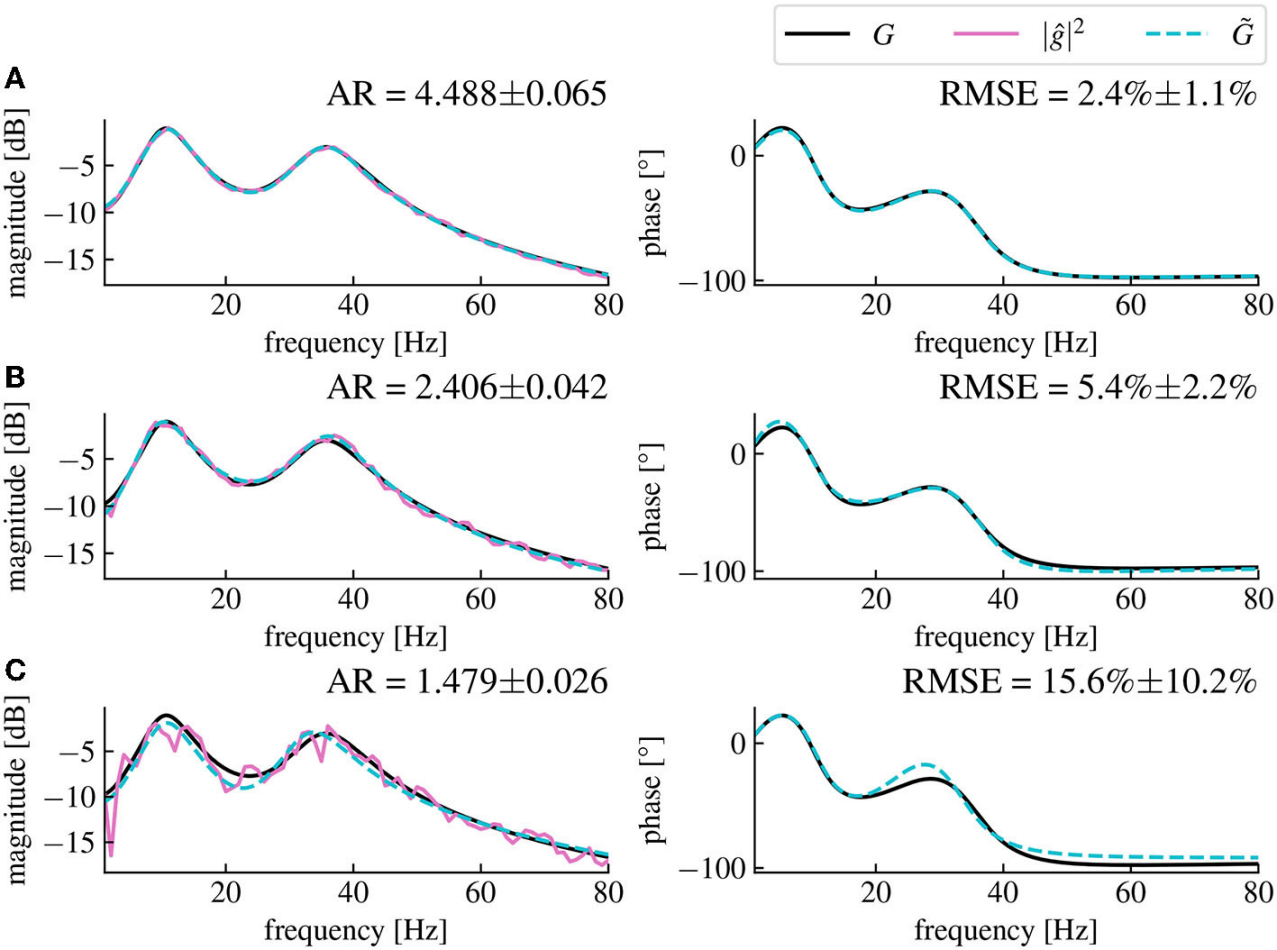
The magnitude vector fitting algorithm's performances depend on the amplitude ratio of the stimulation current and the driving noise. **(A)** Reconstructed magnitude and phase data for an amplitude ratio (AR) of 4.488 ± 0.065. The AR is computed as the ratio between the mean amplitude of *y* and the mean amplitude of *y*_0_ averaged over 50 simulations (CI 95%). The fitted model has a corresponding RMSE of 2.4% ± 1.1% (CI 95%). **(B)** Same as **(A)**, but with an AR of 2.406 ± 0.042, the presented data are the same as in Figures 3C, D. **(C)** Same as **(A)**, but with an AR of 1.479 ± 0.026 resulting in a RMSE of 15.6% ± 10.2% for the fitted model. We see that the noise level in magnitude data is higher for smaller AR, which leads to higher RMSE (CI 95%) between the fitted model's transfer function and the original model's transfer function. The fitted model is coded in dashed cyan and deviates more from the original model for higher noise levels. All data have been computed from 30s long simulated time series.

The remaining error in the estimated model compared to the original model depends on the amplitude ratio between the stimulated output *y* and the resting state output *y*_0_, (cf. Figure 4). Low stimulation currents or high driving noise can also cause the magnitude vector fitting algorithm not to converge, leading to a non-minimal mean-square error between the fitted and the original models when evaluated at the frequency sample points used for the algorithm.

This problem can be solved by increasing the amplitude of the input current *u* that we inject in the plant, which decreases the contribution of the resting state driving noise ξ to the output signal relative to the input current. Although the remaining dominant input current is also noisy, its value at any time or frequency is known, meaning that it is canceled out in the ratio $\frac{S_{yy}}{S_{uu}}$ in Eq. (14). This effectively leads to lower noise in the transfer function magnitude data extracted with Eq. (14). The limitation is then set by the maximum amplitude of the current we are allowed to inject into the brain in a given neurostimulation setup. Indeed, the amplitude of the current is limited both for safety reasons that are beyond the scope of this paper and because of the assumption of linearity on which our method is based and which requires small currents. On the other hand, we can also decrease the noise in the spectral density data by increasing the stimulation time, and hence increasing the amount of data which decreases the contribution of the noise in the power spectral density estimation. Therefore, the accuracy of the model fitting step can be optimized by finding a trade-off between the maximum amplitude of the stimulation current, and the maximum duration of the stimulation.

The accuracy of the fitting is generally easily assessed by computing the root mean square error between the data and the fitted model's transfer function. In practise, this could be used as an indicator to evaluate if sufficient stimulation amplitude and time were chosen and then possibly reiterate this step with different parameters. The root mean square error could also be used to directly quantify the error between the transfer function of the estimated model and the original model, which can be an important parameter regarding the stability and the robustness of the closed-loop while performing delay compensation as discussed in the next section.

### 3.2. Delay compensation

**Figure 5:**
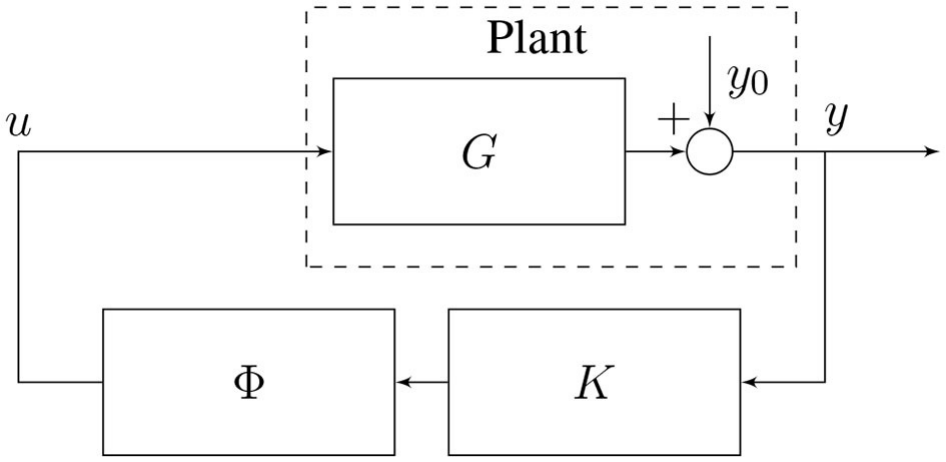
Closed-loop neurostimulation circuit with predictor.

Delay compensation is achieved by adding another LTI system at the output of the controller K (cf. Figure 5), whose purpose is to reproduce the transfer function of a negative delay. We call this system the predictor ϕ.

However, perfectly reproducing the transfer function of a negative delay would be impossible since the associated time-domain system would then be a perfect predictor, which is a non-causal, i.e. un-physical, system. Nonetheless, we can build a causal and stable system that behaves almost like a perfect predictor, however only in the frequency ranges of interest.

The numerical implementation of the controller necessitates discretization in time. Consequently, it is reasonable to choose the predictor design as a discrete-time system, meaning that for any input signal at *x*_*t*_:ℝ → ℝ at an instant *t* ∈ ℝ, it approximately predicts the future signal *x*_*t*+Δ*t*_ where Δ*t* ∈ ℝ is the sampling time chosen when building the predictor. Since *x* is a discrete sequence, its transfer function is obtained using the Z-transform, defined as


$$\begin{array}{l} X\left(z\right)=Z\left\{x_{n\text{Δ}t}\right\}\left(z\right):=\overset{\infty }{\underset{n=0}{\sum }}x_{n\text{Δ}t}z^{-n}, \end{array}$$

with *z* ∈ ℂ and *X*:ℂ → ℂ. Then the transfer function Φ:ℂ → ℂ of a negative delay of one step Δ*t* applied to *x* would simply be Φ(*z*) = *z*, the Z-transform of a one-step delay. However, this choice would be non-causal, which is not implementable numerically in time. Nevertheless, to obtain a stable and implementable system with a transfer function as close as possible to *z*, we chose the ansatz


(15) $$\begin{array}{l} \text{Φ}\left(z_{0}\right)=\frac{b_{0}z_{0}+b_{1}}{z_{0}-a}=z_{0}, \end{array}$$

for a fixed value *z* = *z*_0_ and where *a* ∈ ℝ is the pole of the system and *b*_0_ ∈ ℝ and *b*_1_ ∈ ℝ are the polynomial coefficients of the numerator of Φ. This equation corresponds to the transfer function of a discrete LTI system with exactly one pole and one zero, which is the closest form of a proper rational function to the identity function of *z* in the sense that it has only one more pole. We add the additional constraints that |*a*| < 1, since this is the necessary and sufficient condition for the discrete predictor ϕ to be stable.

We choose to reformulate this problem by setting *a* as a free parameter. This way, we can select any *a* between −1 and 1, and the remaining parameters are found by solving the linear equation *b*_0_*z*_0_+*b*_1_ = *z*_0_(*z*_0_−*a*), where *z* ∈ ℂ is a chosen complex frequency point at which we want this equation to hold. Since there are two unknowns, we can write a second equation in which we want the derivative of each side of the equation also to be equal, yielding *b*_0_ = 2*z*_0_−*a*. By replacing *b*_0_ in the first equation, we obtain


$$\begin{array}{l} z_{0}(2z_{0}-a)+b_{1}=z_{0}(z_{0}-a)\\ \;b_{1}\text{ =}-z_{0}^{2}. \end{array}$$

In the z-domain, the zero frequency corresponds to *z*_0_ = 1. We choose to solve this equation for this point, hence we can replace *a*, *b*_0_ and *b*_1_ in Eq. (15) which yields


(16) $$\begin{array}{l} \text{Φ}\left(z\right)=\frac{\left(2-a\right)z-1}{z-a}. \end{array}$$

This transfer function can then be converted to an associated state-space representation and used for time domain simulations with a sampling time Δ*t*. The output of this system will then be *y*_*t*_≈*u*_*t*+Δ*t*_ for any input signal *u*_*t*_. Simulating delays greater than the system sampling time is simply achieved by concatenating multiple times this predictor system. Here the delay has to be a multiple of the sampling time. This predictor can then be appended to the output of the digital controller K.

**Figure 6:**
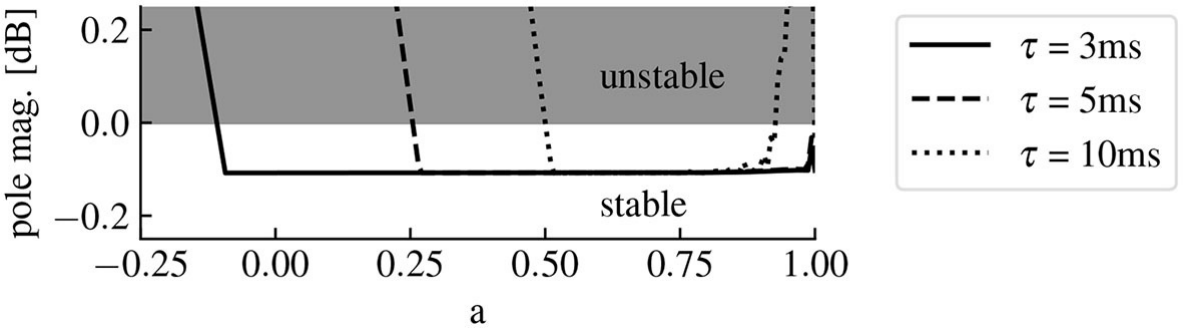
The predictor pole location affects the closed-loop stability. The magnitude of the pole with the highest magnitude in the closed-loop transfer function parameterizes the stability of the closed-loop. Indeed, if this value is less than 0 dB, then all the poles of the closed-loop transfer function have a magnitude less than 0 dB, meaning that the system is stable. The system is unstable otherwise. Here the full curve, the dashed curve and the dotted curve correspond to predictors for delays of 3 ms, 5 ms and 10 ms, respectively. The higher the delay is, the lower is the size of the region of closed-loop stability for *a*.

To avoid closed-loop instability, we must limit the amplitude of the feedback signal computed from the controller input signal. This amplitude is determined by the three systems G, H and K. Since G is defined by the system under study and H is the chosen filter defining the desired modifications in the frequency distribution of the observed signal, ϕ (or equivalently parameter *a*) is the only degree of freedom. Figure 6 shows the region of closed-loop stability as a function of the predictor pole *a* and the delay.

Because the predictor has a gain that is still slightly greater than one in the frequency ranges of interest, we reduce the weights of the filter H to compensate for the excess gain at the α and γ-peaks. To do this, we simply divide the weight of each band by the magnitude of the predictor system evaluated at the band's natural frequency. This reduces the errors in the closed-loop transfer function in the α and γ-ranges.

**Figure 7:**
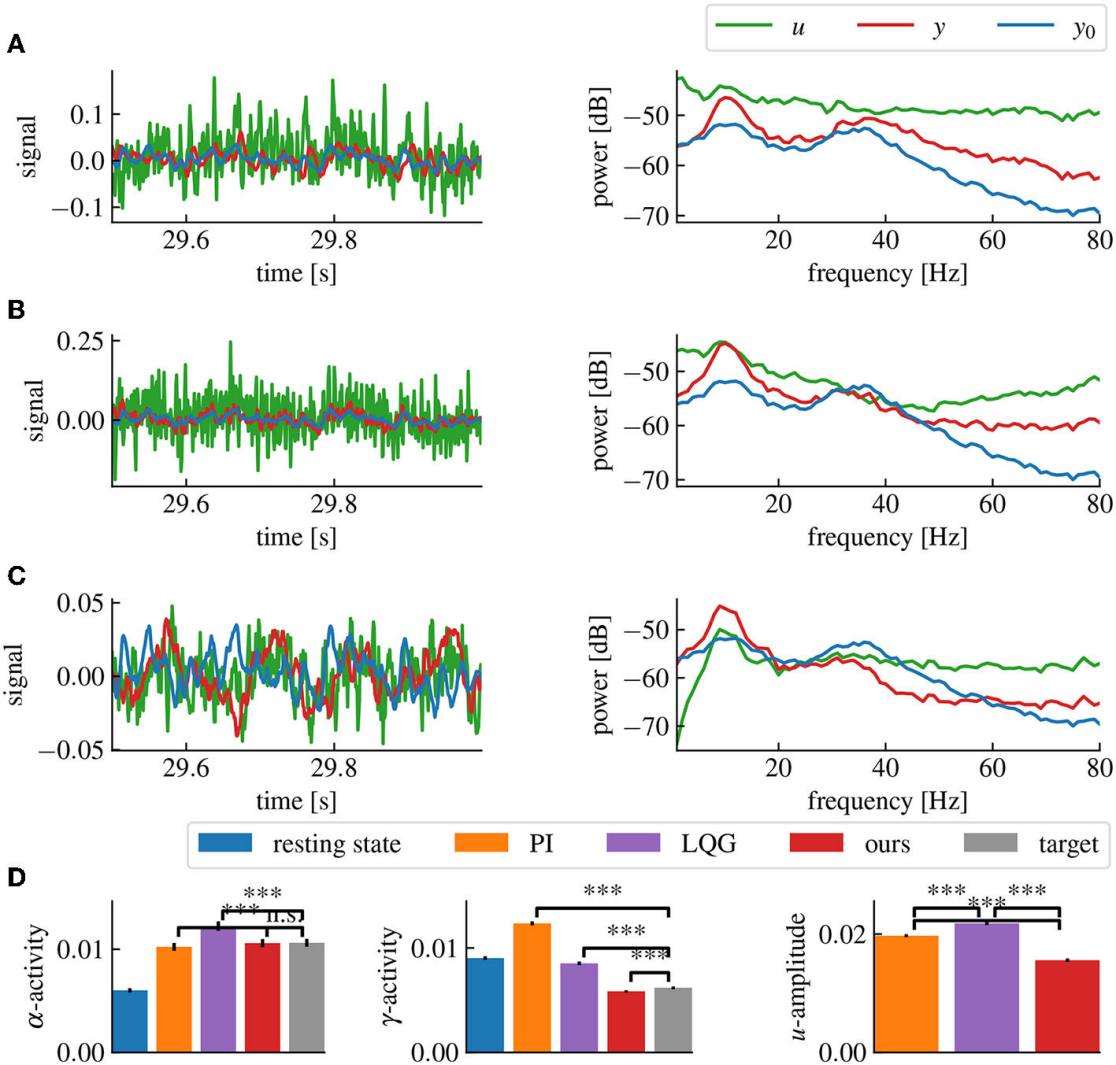
Our method successfully decreases α- and γ-activities in the presence of a 5ms delay, while maintaining a low stimulation current. **(A)** Simulation time series (left panel) and power spectral densities (right panel) of the PI control loop with Smith predictor. **(B)** Same as **(A)** for the LQG control loop with Smith predictor. **(C)** Same as **(A)** for our method, including delay compensation. **(D)** α-, γ-activities and mean amplitude of *u* for each method, averaged over 50 simulations. “***” corresponds to a *p*-value less than 0.05 with Welch's *t*-test, while “n.s.” correspond to a *p*-value higher than 0.05. Our method provides both the closest match to the target α- and γ-activities and the lowest stimulation current (*u*) amplitude. The parameters for all controllers have been chosen to match the target activities as closely as possible without destabilizing the closed-loop. The activities are computed by averaging the spectral densities in their corresponding ranges while the *u*-amplitude correspond to the average spectral density of *u* from 0Hz to the Nyquist frequency.

Figure 7C shows results combining the model estimation by vector fitting and the delay compensation. The proposed closed-loop control yields an increase in α-power and a decrease in γ-power according to the employed target filter H. The application of PI and LQG control with a Smith predictor for delay compensation (Figures 7A, B) has poor performances for higher γ-frequency activity.

#### 3.2.1. Accuracy

**Figure 8:**
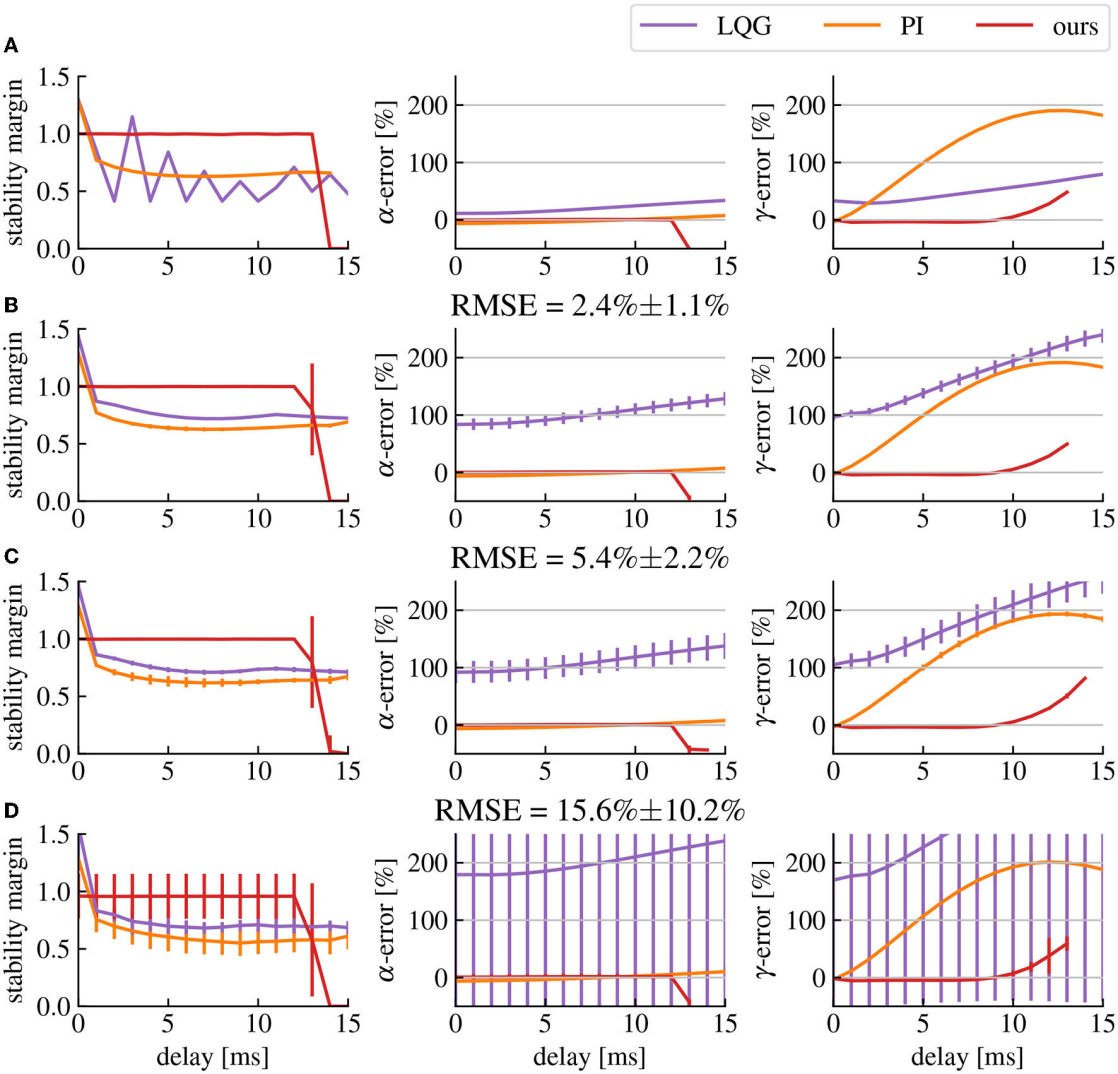
Delay affects the stability and robustness of the closed-loop transfer function. **(A)** Stability margin and α- and γ-activities errors in function of the delay with no model errors. The stability margin is computed as the shortest distance between the Nyquist plot and the point −1. The higher the stability margin, the more robust the closed-loop is to model uncertainties. The α- and γ-errors represent the error percentage between the measured activity and the target activity. **(B–D)** Same as row **(A)** but with model errors (quantified as the RMSE of the model fitting step) corresponding respectively to the model fits shown in Figures 4A–C. The vertical bars represent the standard deviation of their corresponding point.

The error between the achieved closed-loop output activity levels and the target activity levels is highly affected by the delay (cf. Figure 8). This is caused by the phase shift between the input and the output signal, which changes the effect of the control signal on the output in a frequency dependent manner. Our frequency range of interest is limited to frequencies below 55 Hz, which is the higher limit we use for the γ-range. In this case, the effect of delays of the order of milliseconds will be more visible for higher frequencies and higher delays (cf. Figure 8). However, using our predictor design, we significantly mitigate the effects of the delay in the frequency ranges of interest (cf. Figure 8) red curve. Nonetheless, these effect are still present, creating a limit to the maximum delay our predictor is able to compensate, which in our case is situated around 10ms.

#### 3.2.2. Stability and robustness

As discussed earlier, delay compensation can destabilize the closed-loop system depending on the parameters of its components. However, if the correct predictor pole is chosen based on Figure 6, the closed-loop will remain stable. These values are computed under the assumption that there are no model estimation errors. If we take into account the inaccuracies in the fitted brain model compared to the original brain model, extra gain can add up in the feedback signal, introducing again the risk of destabilizing the closed-loop (cf. Figure 8). The solution is either to simply reduce the amplitude of the spectral density modification that we want to apply by reducing the amplitude of the transfer function of filter H, or to reduce the amplitude of the predictor Φ reducing its accuracy and possibly increasing delay errors. There is then a trade-off between how much we want to change the gain of the closed-loop transfer function while also compensating delay errors and how much we want to avoid closed-loop destabilization caused by model uncertainties. In any case, the inaccuracies in the estimated brain model create errors in the closed-loop transfer function regardless of the delay, which makes them the main determinant of the performance limits of our method in any given setup. Nevertheless, our method produces smaller α- γ-activities errors than LQG control and produces the smallest error for γ-activities for delays of 1 to 12ms (cf. Figure 8). Comparisons lead to *p*-values less than 0.0005 using Welch's *t*-test, except for LQG control in row D where the variance of the data was to high to find any significant difference with this test.

### 3.3. Application to cortico-thalamic circuit model

**Figure 9:**
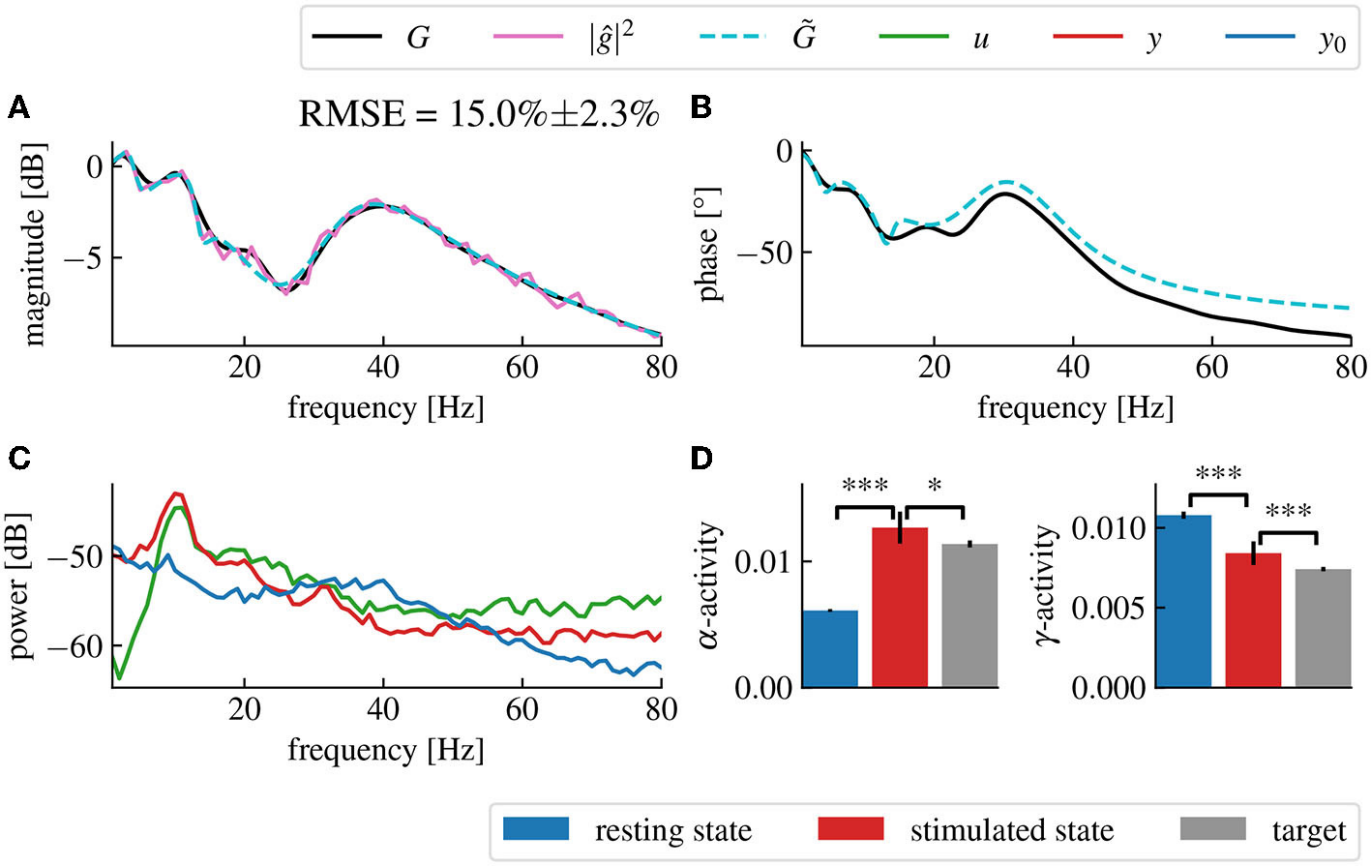
Fitted model-based control using the cortico-thalamic brain model successfully reproduces the target transfer function in the frequency domains of interest. **(A)** Magnitude of the fitted brain model transfer function (dashed cyan) obtained from the power spectral density data (pink) compared to the magnitude of the original cortico-thalamic brain model transfer function (black). **(B)** Phase shift of the fitted transfer function (dashed cyan) compared to the phase shift of the original transfer function (black). **(C)** Spectral densities of the resting state activity signal (blue), the stimulated brain output (red) and the stimulation signal (green). **(D)** α- and γ-activities of the closed-loop output averaged over 50 trials for which the fitting step was repeated each time. “***” corresponds to a *p*-value less than 0.0005 with Welch's *t*-test while “*” corresponds to a *p*-value less than 0.05. The α- and γ-activities are respectively increased and decreased after application of the closed-loop.

To extend the analysis to a biologically more realistic model, we employed a nonlinear cortico-thalamic brain model (cf. Section 2.7.2). Fitting a linear transfer function to the brain model activity as described above, we found a good accordance of fitted and original model as can be seen in Figures 9A, B. Small deviations in the gain and the phase resulted from the internal delay in the brain model and its non-linearity. Indeed, the magnitude vector fitting algorithm does not reproduce this delay but instead synthesizes a linear system that has no delay but still approximates well the transfer function of the original model. Nonetheless, the non-linearity of this model can also decrease the accuracy of the fitting, as we are trying to represent a non-linear input response model by a linear one. However, this effect is only seen when the current is large enough for the non-linear part of the response to be significant.

In fact, the model-based control enhances α-activity and diminishes γ-activity in good accordance to the imposed filter H (Figure 9C). The closed-loop transfer function deviates from the target transfer function for large frequencies beyond the γ-frequency range. This results from the employed conduction delay.

**Figure 10:**
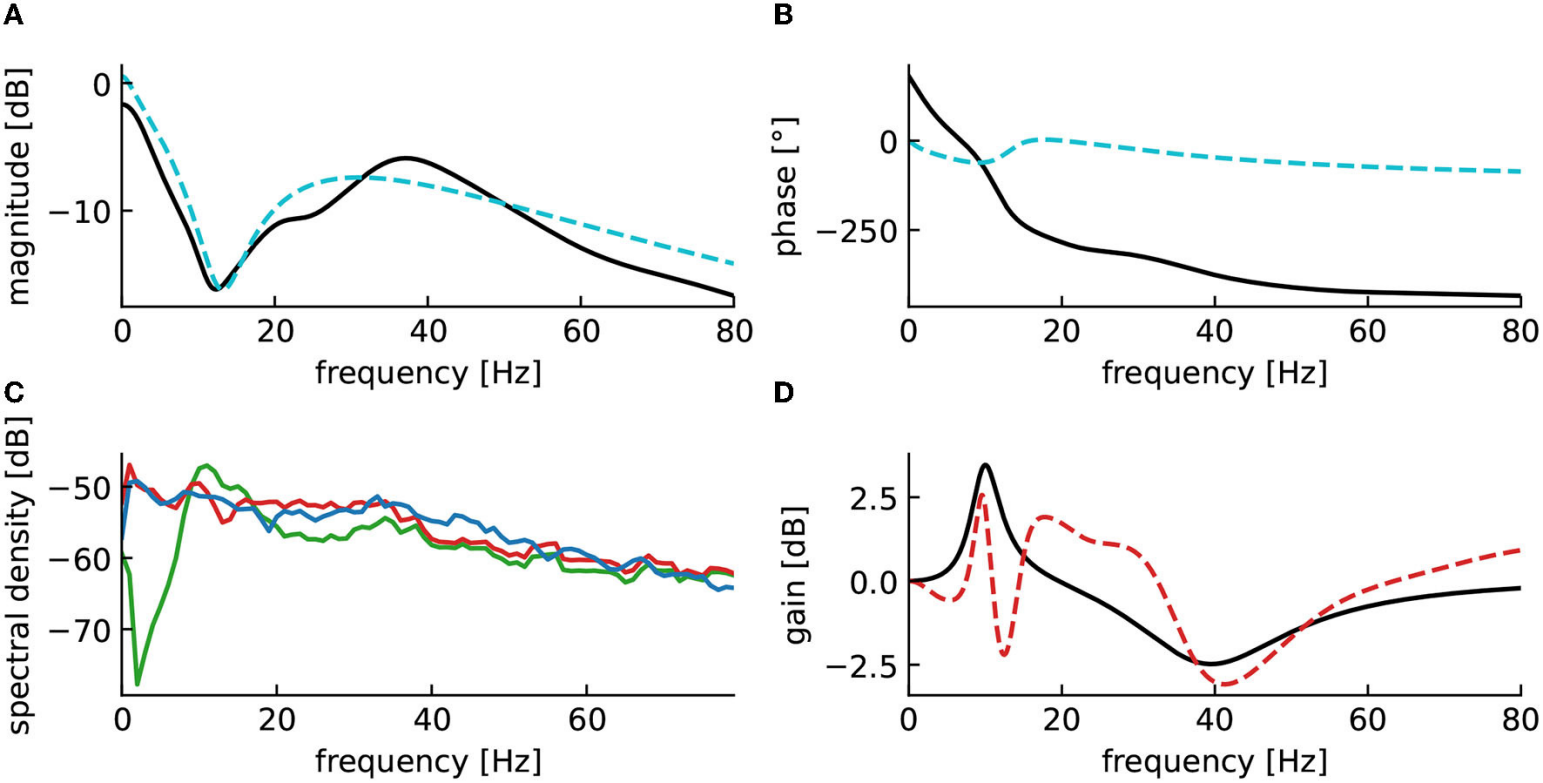
Reticulum stimulation yields incorrect closed-loop gain in low-frequency ranges. **(A)** Magnitude of the fitted brain model transfer function (dashed cyan) compared to the magnitude of the original cortico-thalamic brain model transfer function (black). **(B)** Phase shift of the fitted transfer function (dashed cyan) compared to the magnitude of the original transfer function (black). **(C)** Spectral densities of the resting state activity signal (blue), the stimulated brain output (red) and the stimulation signal (green). **(D)** Closed-loop transfer function (dashed red), compared to the target transfer function 1 + *H*(*s*) (black).

To elucidate better the functions of the different elements of the proposed method, we applied a second closed-loop setup, where the neurostimulation input was applied to the first three layers of the cortex modeled by *u* and *v* and to the reticulum modeled by *V*_*ret*_ (Figure 10). In this setting, the response in the high-frequency ranges are mainly produced by the cortex, while the response in low-frequency ranges originates mainly from the reticulum and the thalamic relay structure, with a gap approximately between 10Hz and 20Hz. The weak response between 10 and 20 Hz observable (cf. Figure 10A) is compensated by the controller, which produces a high magnitude stimulation in the closed-loop for these frequencies (cf. Figure 10C). The second consequence is the inaccuracy of the closed-loop output in the low-frequency ranges, this is caused by the rather long cortico-thalamic internal delay. This delay yields a larger phase shift at low-frequencies and originates from the fact that we observe signals in the cortex, but stimulate in the reticulum.

## 4. Discussion

The goal of the proposed method was to design a delayed closed-loop control method to apply defined modifications to the spectral distribution of an observed signal, such as EEG or LFP. Under the assumption of linear brain stimulation response, the presented work explicitly describes all the steps needed to build a delayed closed-loop neurostimulation setup to restore the physiological brain state of a patient (Hebb et al., 2014). Since the controller is modeled as a linear time-invariant system, its implementation is lightweight, straightforward, and easily applicable in most embedded systems. Applications to a simple neural populations model (Figure 7) and to a biologically plausible cortico-thalamic feedback system (Figures 9, 10) demonstrate its elements and their impact on the control performance.

### 4.1. Main contributions

Our method allows to precisely specify the desired frequency-domain modifications we want to apply to the brain activity. The resulting closed-loop controller can then synthesize in real-time the required closed-loop neurostimulation signal necessary to reach the desired output, without the need to track a pre-defined reference signal. This make the method more flexible since it requires to specify relative rather that absolute signal modifications which is preferable considering the intra- and inter-patients variability of the EEG spectrum. Furthermore, specifying a reference signal which is a stochastic signal uncorrelated with the noise of the current plant output introduces additional noise in the feedback signal, since the controller needs to compensate for both the mean difference between the frequency distributions of the two signal and the difference between the driving noises of the two signals. Therefore, our method is able to track a target frequency distribution for the brain output with a lower current amplitude than classical methods (cf. Figure 7).

#### 4.1.1. Model estimation

We assume resting state activity signal driven by noise, when no neurostimulation is applied. Injecting a stimulation creates an additional response that adds to the resting state. Consequently, both the resting state signal and response signal can be observed separately in experimental practice and they serve to estimate a linear state-space model as outlined in Section 3.1. This approach is successful for both simplified linear models (cf. Figures 3, 4) and neurophsysiological realistic nonlinear models (cf. Figure 9). This approximation is suitable for nonlinear systems whose dynamics evolve close to a stationary state. Several studies have already exposed evidence confirming that the measured brain dynamics behave mostly linearly at macroscopic scales (Popivanov et al., 1996; Liu et al., 2010). Moreover, in the case of the brain response to small neurostimulation input, our assumption of the linear brain response is supported by results of Kim and Ching (2016). The authors of this study measured the controllability Gramian of their brain model with nonlinear sigmoid transfer function, similar to the cortico-thalamic brain model (Riedinger and Hutt, 2022) used in this paper. If the system exhibits nonlinear dynamics far from any linear approximation, such as bistable dynamics and chaotic evolution, the proposed vector fitting technique may yield a too large model error and thus instability of the closed-loop feedback. The hypothesis of macroscopically linear dynamics has also recently been tested against various nonlinear models (Nozari et al., 2020). While that work included fitting methods for both linear and nonlinear brain models, our work chose the paradigm of purely frequency domain model fitting with the magnitude vector fitting algorithm (De Tommasi et al., 2010) and applied it to the brain input response system, which we could isolate thanks to a simple open-loop neurostimulation setup. While models have already been studied in application to neurostimulation (Modolo et al., 2011; Wagner et al., 2014), we propose a straightforward black box modeling approach that is directly usable for adaptive closed-loop neurostimulation, and is technically applicable easily for each individual patients before any closed-loop neurostimulation sessions.

#### 4.1.2. Delay compensation

Conduction delays of a few milliseconds in the transmission between observation and stimulation may be negligible in systems evolving on time scales of seconds or longer, but may play an important role in neural systems. Our study demonstrates that such feedback delays may introduce control errors and we show how these errors can be avoided by a novel delay compensation method (Section 3.2). Application to the linear model (7) demonstrated its superior performance compared to conventional delay compensation methods. Delay compensating systems have already been described in other work (Guo et al., 2004; Hosseini et al., 2019). However, we used a design primarily focused on the correction of a gain error in the closed-loop transfer function, whereas the majority of the current research is based on time domain criterion and stability enforcement (Sönmez and Ayasun, 2015; Ledva et al., 2017). The methods performance, i.e. how well the total gain function fits to the pre-defined transfer function, is good for low-frequencies but weakens for frequencies exceeding a limit frequency. Note that frequency domain compensation has also already been achieved, notably via delay equalizers (Podilchak et al., 2009). However, this would restrict the frequency range in which the delay is compensated, and create additional errors in the surrounding frequencies. Other designs include filters with negative group delays, however their applications are limited to band limited input signals (Bukhman and Bukhman, 2004; Voss, 2017). The predictor design we presented also relies on negative group delay, enabling delay compensation in a large frequency band, while still being applicable to the brain EEG, which is inherently not band limited, because of the noise. Nonetheless, while our predictor design allows to significantly decreases the delay errors in the closed-loop transfer function, the delay still imposes a limit on the controllable frequency range. The larger the delay, the smaller is this limit frequency. Low performance may induce instability in the feedback loop (Mirkin and Palmor, 2005) and thus should be avoided. A corresponding stability criteria has been proposed, (cf. Figure 6). Better predictor designs could allow better performance of the closed-loop system for larger delays. The improvement of the accuracy of our closed-loop neurostimulation setup by building more efficient predictor designs is in progress and we refer the reader to future work.

### 4.2. Limits of our methodology

#### 4.2.1. Experimental stimulation parameters and safety

Experimental stimulation protocols have to ensure the subjects safety (Ko, 2021) and thus avoide stimulus-induced health risks and complications. For instance, tDCS may be administered for a duration of 60 minutes and a maximum current of 4 mA without yielding health risks. However, parameters beyond these limits may yield adverse effects in subjects, such as skin lesions similar to burns and mania or hypomania in patients with depression (Matsumoto and Ugawa, 2017). The proposed method does not limit the stimulation duration *per se*, but of course the duration can be chosen accordingly without constrating the method. The method adapts the systems brain rhythms to the target rhythms very rapidly on a time scale of less than a second and hence permits rather short stimulation duration longer than a second.

Moreover, the proposed method does not specify absolute stimulation current magnitude applied. The impact of stimulation at certain magnitudes depends heavily on the stimulation type. In tDCS, anodal stimulation with positive currents have a different impact as cathodal stimulation with negative currents. In addition, currents are thought to have to pass a certain threshold to yield a measurable effect. In tACS (Moliadze et al., 2012), stimulating in the α-frequency range large and small magnitudes yield excitation and inhibition, respectively, while intermediate magnitudes yield weak effects. Stimulating with a range of frequencies, as in tRNS (Potok et al., 2022), a 1mA peak-to-peak amplitude for 10 minutes stimulation duration does not yield adverse effects. We conclude that it is not straight-forward to decide which stimulation magnitude applied in the presented method would be safe for human subjects, since the stimulation signal is neither constant, single frequency oscillation nor random noise. In sum, we argue that a maximum peak-to-peak amplitude of 1mA for few tens of minutes may not yield adverse effects, but still may evoke a measurable impact on observations and the brain state. Of course, future experimental studies will gain deeper insights.

#### 4.2.2. Model internal delay

The internal delay in the brain is not reproducible by the magnitude vector fitting algorithm, which relies on the time invariance of the signals. Hence, this will cause errors in the transfer function of the fitted model (cf. Figure 9) that are larger for higher contribution of the delay in the output (cf. Figure 10). To limit this effect, we must minimize the delay between the application of the neurostimulation input and the measurement of the response to this input as much as possible by taking into account the delay between the different brain regions.

#### 4.2.3. Estimating the closed-loop delay

For delay compensation, in this paper, we assumed that we know the conduction delay in the closed-loop. However, although it is a single constant parameter, we would need a method to measure it for a real closed-loop neurostimulation setup. A straightforward way to do this would be to inject any current into the plant and measure the time lag between the moment at which we inject the input current and the moment at which we measure the output signal. This estimated delay would then correspond to the total closed-loop delay except for the computation delay of the digital controller K. This computation delay can be easily measured with the same software used for computation, as it corresponds to the delay needed to perform constant-size matrix multiplications. Moreover, several methods have already been developed to estimate the conduction delays in linear systems (Schier, 1997; Dudarenko et al., 2014).

#### 4.2.4. Direct input current measurements

One of the main challenges to solve for closed-loop neurostimulation is the elimination of direct transmission artifacts from the measured EEG signal (Iturrate et al., 2018). Indeed, when measuring the plant output signal, a portion of the measured signal might be a direct measurement of the input current without any influence from the brain dynamics. In the ideal case, one intends to minimize the contribution of the stimulation input to the observed signal since it would mean that the measured EEG signal does not fully correspond to the brain activity. Hence, reading the EEG of the patient would be more difficult for the user of our closed-loop setup, and the contribution of the brain dynamics to the closed-loop would be smaller. A simple solution to this problem is discussed further below.

### 4.3. Perspectives

The control proposed allows to perform accurate frequency shaping of the systems' activity spectral distribution. However, this approach is limited to linear models of the brain stimulation response. This may be disadvantageous if the systems dynamics exhibit nonlinear behavior (see e.g., Hutt and beim Graben, 2017) as we want to represent the brain dynamics realistically. Furthermore, in real-case scenarios, we would also have to take into account the noise in the acquisition of the signal by the sensor and in the application of the input signal by the actuator.

#### 4.3.1. Filtering out direct input current measurements

Filtering out the direct input current measurements is achievable with our setup removing the strictly proper system requirement while using the magnitude vector fitting algorithm to measure the brain input response. In other words, while fitting the brain input response system, we want the fitted model to be able to contain a direct transmission term corresponding to the direct current measurement. Hence, if the real plant input response contains a significant direct transmission term, it will be identified by the magnitude vector fitting algorithm when synthesizing the estimated plant input response. The second step is them simply to substract the feedtrough term multiplied by the input current to the plant output signal. Thus, the remaining part of the signal would only correspond to the brain dynamics.

#### 4.3.2. Application to multiple inputs multiple outputs plants

For now, we only focused on plant with a signal input signal and a single output signal. However, in a real setup, the EEG measurement is typically composed of multiple channels corresponding to different electrodes. This can also be true for the neurostimulation device. For example, with electric current stimulation, we can inject multiple signals using multiple electrodes. This can be simply solved by feeding a single input to each input channel and summing each output to a single output channel. However, when we separate the different channels, we can have more control over each individuals output channels. When we have multiple inputs and output, the plant is then a Multiple-Inputs Multiple-Outputs (MIMO) system. Everything developed in this paper is generalizable to MIMO systems, with one caveat: when solving E.q., (6), a unique solution only exists if the system has as more outputs than it has inputs. The user can always ensure this, by using as many neurostimulation input channels than there are EEG output channels. In this generalized setup, we can also define the filter H to apply different modifications to each output channel.

#### 4.3.3. Neurostimulation effects on larger time scales

Our method relies only on the short term dynamics of the brain, using signal feedback and delay compensation to produce an adaptive stimulation current and obtain the desired EEG frequency distribution. However, more traditional neurostimulation techniques rely on the long term dynamics of neural plasticity, which is not modeled in the brain models we use in this paper. Long term brain adaptation to neurostimulation could cause the EEG frequency distribution to diverge from the desired frequency distribution after several minutes of stimulation. This effect could be compensated either by reiterating the model identification step and performing neurostimulation again, or by adjusting the weight of the filter H according to the observed changes in real-time. Incorporating the effect of neural plasticity in the brain models would allow our method to produce predictable and durable modification to the EEG frequency distribution, even after we stop the stimulation.

## Data availability statement

The source code used for the simulation results can be freely accessed here: https://github.com/Thomas-Wahl/neuroclodec.

## Author contributions

TW, AH, and MD contributed to the development of the methods presented in this study. TW produced the source code used for the simulations. JR and AH wrote the introduction section. TW and AH wrote the other sections of the manuscript. All authors read and approved the submitted version.

## References

[B1] Åström K. J. (2012). Introduction to Stochastic Control Theory. Chelmsford, MA: Courier Corporation.

[B2] Basar E. (2013). Brain oscillations in neuropsychiatric disease. Dialogues Clin. Neurosci. 15, 291–300. 10.31887/DCNS.2013.15.3/ebasar 24174901 PMC3811101

[B3] Bennabi D. Haffen E. (2018). Transcranial direct current stimulation (tdcs): a promising treatment for major depressive disorder? Brain Sci. 8, 81. 10.3390/brainsci8050081 29734768 PMC5977072

[B4] Bolus M. F. Willats A. A. Rozell C. J. Stanley G. B. (2021). State-space optimal feedback control of optogenetically driven neural activity. J. Neural Eng. 18, 036006. 10.1088/1741-2552/abb89c 32932241 PMC8356067

[B5] Bolus M. F. Willats A. A. Whitmire C. J. Rozell C. J. Stanley G. B. (2018). Design strategies for dynamic closed-loop optogenetic neurocontrol *in vivo*. *J. Neural Eng*. 15, 026011. 10.1088/1741-2552/aaa506 29300002 PMC5957547

[B6] Brunelin J. Mondino M. Gassab L. Haesebaert F. Gaha L. Suaud-Chagny M.. (2012). Examining transcranial direct-current stimulation (tDCS) as a treatment for hallucinations in schizophrenia. Am. J. Psychiatry. 169, 719–724. 10.1176/appi.ajp.2012.11071091 22581236

[B7] Bukhman N. Bukhman S. (2004). On the negative delay time of a narrow-band signal as it passes through the resonant filter of absorption. Radiophys. Quantum Electron. 47, 68–76. 10.1023/B:RAQE.0000031672.70934.3a

[B8] Chen Z. S. Kulkarni P. P. Galatzer-Levy I. R. Bigio B. Nasca C. Zhang Y. (2022). Modern views of machine learning for precision psychiatry. Patterns 3,100602. 10.1016/j.patter.2022.100602 36419447 PMC9676543

[B9] De Tommasi L. Gustavsen B. Dhaene T. (2010). Robust transfer function identification via an enhanced magnitude vector fitting algorithm. IET Control Theory & *Applicat*. 4, 1169–1178. 10.1049/iet-cta.2009.0025

[B10] Dudarenko N. Polinova N. Ushakov A. (2014). Fundamental matrix of linear continuous system in the problem of estimating its transport delay. Nauchno-Tekhnicheskii Vestnik Informatsionnykh Tekhnologii, Mekhaniki i Optiki. 14, 5.

[B11] Edel Y. Caroli F. (1987). Histoire de l'électrochoc : des traitements électriques à la convulsivothérapie en psychiatrie. Bulletin d'histoire de l'électricité. 9, 87–114. 10.3406/helec.1987.1012

[B12] Fang H. Yang Y. (2022). Designing and validating a robust adaptive neuromodulation algorithm for closed-loop control of brain states. J. Neural Eng. 19, 036018. 10.1088/1741-2552/ac7005 35576912

[B13] Fang H. Yang Y. (2023). Predictive neuromodulation of cingulo-frontal neural dynamics in major depressive disorder using a brain-computer interface system: A simulation study. Front. Comput. Neurosci. 17, 1119685. 10.3389/fncom.2023.1119685 36950505 PMC10025398

[B14] Fleming J. Dunn E. Lowery M. (2020). Simulation of closed-loop deep brain stimulation control schemes for suppression of pathological beta oscillations in parkinson's disease. Front. Neurosci. 14, 166. 10.3389/fnins.2020.00166 32194372 PMC7066305

[B15] Gardiner C. (2004). Handbook of Stochastic Methods. Berlin: Springer. 10.1007/978-3-662-05389-8

[B16] Guerrero Moreno J. Biazoli J.r. C Fontes Baptista A. Remoaldo Trambaiolli L. (2021). Closed-loop neurostimulation for affective symptoms and disorders: an overview. Biol. Psychol., 161:108081. 10.1016/j.biopsycho.2021.108081 33757806

[B17] Guo L. Cardullo F. Houck J. Kelley L. Wolters T. (2004). “New predictive filters for compensating the transport delay on a flight simulator,” in AIAA Modeling and Simulation Technologies Conference and Exhibit (American Institute of Aeronautics and Astronautics). p. 5441. 10.2514/6.2004-5441

[B18] Haeusermann T. Lechner C. Fong K. Sideman A. Jaworska A. Chiong W.. (2023). Closed-loop neuromodulation and self-perception in clinical treatment of refractory epilepsy. AJOB Neurosci. 14, 32–44. 10.1080/21507740.2021.1958100 34473932 PMC9007331

[B19] Hartshorn A. Jobst B. (2018). Responsive brain stimulation in epilepsy. Ther. Adv. Chronic Dis. 9, 135–142. 10.1177/2040622318774173 29963302 PMC6009082

[B20] Hashemi M. Hutt A. Sleigh J. (2015). How the cortico-thalamic feedback affects the EEG power spectrum over frontal and occipital regions during propofol-induced anaesthetic sedation. J. Comput. Neurosci. 39, 155. 10.1007/s10827-015-0569-1 26256583

[B21] Hebb A. O. Zhang J. J. Mahoor M. H. Tsiokos C. Matlack C. Chizeck H. J.. (2014). Creating the feedback loop: closed-loop neurostimulation. Neurosurg. Clini. 25, 187–204. 10.1016/j.nec.2013.08.006 24262909 PMC4058859

[B22] Hespanha J. P. (2018). Linear Systems Theory. Princeton: Princeton University Press. 10.23943/9781400890088

[B23] Hirano Y. Orbie N. Kanba S. Onitsuka T. Nestor P. Spencer K. (2015). Spontaneous gamma activity in schizophrenia. JAMA Psychiat. 72, 813–821. 10.1001/jamapsychiatry.2014.2642 25587799 PMC4768724

[B24] Holtzheimer P. Mayberg H. (2011). Deep brain stimulation for psychiatric disorders. Annu. Rev. Neurosci. 34, 2890307. 10.1146/annurev-neuro-061010-113638 21692660 PMC4413475

[B25] Hosain M. Kouzani A. Tye S. (2014). Closed loop deep brain stimulation: an evolving technology. Australas. Phys. Eng. Sci. Med. 37, 619–634. 10.1007/s13246-014-0297-2 25195055

[B26] Hosseini S. A. Toulabi M. Dobakhshari A. S. Ashouri-Zadeh A. Ranjbar A. M. (2019). Delay compensation of demand response and adaptive disturbance rejection applied to power system frequency control. IEEE Transac. Power Syst. 35, 2037–2046. 10.1109/TPWRS.2019.2957125

[B27] Howells F. Temmingh H. Hsieh J. van Dijen A. Baldwin D. Stein D. (2018). Electroencephalographic delta/alpha frequency activity differentiates psychotic disorders: a study of schizophrenia, bipolar disorder and methamphetamine-induced psychotic disorder. Transl. Psychiatry. 8:75. 10.1038/s41398-018-0105-y 29643331 PMC5895848

[B28] Hutt A. (2013). The anesthetic propofol shifts the frequency of maximum spectral power in eeg during general anesthesia: analytical insights from a linear model. Front. Comput. Neurosci. 7, 2. 10.3389/fncom.2013.00002 23386826 PMC3564209

[B29] Hutt A. beim Graben P. (2017). Sequences by metastable attractors: interweaving dynamical systems and experimental data. Front. Appl. DYn. Syst. Stat. 3, 11. 10.3389/fams.2017.00011

[B30] Iturrate I. Pereira M. Millán J. R. (2018). Closed-loop electrical neurostimulation: challenges and opportunities. Curr. Opini. Biomed. Eng. 8, 28–37. 10.1016/j.cobme.2018.09.00731572109

[B31] Khintchine A. (1934). Korrelationstheorie der stationären stochastischen prozesse. Mathematische Annalen. 109, 604–615. 10.1007/BF01449156

[B32] Kim S. A. Ching S. (2016). “Quasilinearization-based controllability analysis of neuronal rate networks,” in 2016 American Control Conference (ACC). Boston, MA: IEEE. p. 7371–7376.

[B33] Ko M. (2021). Safety of transcranial direct current stimulation in neurorehabilitation. Brain Neurorehabil. 14, e9. 10.12786/bn.2021.14.e9 36742105 PMC9879413

[B34] Kühn A. A. Kempf F. Brücke C. Gaynor Doyle L. Martinez-Torres I. Pogosyan A.. (2008). High-frequency stimulation of the subthalamic nucleus suppresses oscillatory β activity in patients with parkinson's disease in parallel with improvement in motor performance. J. Neurosci. 28, 6165–6173. 10.1523/JNEUROSCI.0282-08.2008 18550758 PMC6670522

[B35] Ledva G. S. Vrettos E. Mastellone S. Andersson G. Mathieu J. L. (2017). Managing communication delays and model error in demand response for frequency regulation. IEEE Transact. Power Syst. 33, 1299–1308. 10.1109/TPWRS.2017.2725834

[B36] Leicht G. Vauth S. Polomac N. Andreou C. Rauh J. Mußmann M.. (2015). EEG-Informed fMRI reveals a disturbed gamma-band–specific network in subjects at high risk for psychosis. Schizophr. Bull. 42, 239–249. 10.1093/schbul/sbv092 26163477 PMC4681551

[B37] Liu Z. Rios C. Zhang N. Yang L. Chen W. He B. (2010). Linear and nonlinear relationships between visual stimuli, eeg and bold fmri signals. Neuroimage. 50, 1054–1066. 10.1016/j.neuroimage.2010.01.017 20079854 PMC2841568

[B38] Martin S. Iturrate I. Chavarriaga R. Leeb R. Sobolewski A. Li A.. (2018). Differential contributions of subthalamic beta rhythms and 1/f broadband activity to motor symptoms in parkinson's disease. NPJ Parkinson's Dis. 4, 32. 10.1038/s41531-018-0068-y 30417084 PMC6218479

[B39] Matsumoto H. Ugawa Y. (2017). Adverse events of tdcs and tacs: a review. Clini. Neurophysiol. Pract. 2, 19–25. 10.1016/j.cnp.2016.12.003 30214966 PMC6123849

[B40] Mirkin L. Palmor Z. J. (2005). Control Issues in Systems with Loop Delays, chapter 59. Boston, MA: Birkhäuser Boston. p. 627–648.

[B41] Modolo J. Legros A. Thomas A. W. Beuter A. (2011). Model-driven therapeutic treatment of neurological disorders: reshaping brain rhythms with neuromodulation. Interface Focus. 1, 61–74. 10.1098/rsfs.2010.0509 22419974 PMC3262241

[B42] Moliadze V. Atalay D. Antal A. Paulus W. (2012). Close to threshold transcranial electrical stimulation preferentially activates inhibitory networks before switching to excitation with higher intensities. Brain Stimul. 5, 505–511. 10.1016/j.brs.2011.11.004 22445135

[B43] Morari M. Zafiriou E. (1989). Robust Process Control. Madison, WI: Morari.

[B44] Nasr K. Haslacher D. Dayan E. Censor N. Cohen L. G. Soekadar S. R. (2022). Breaking the boundaries of interacting with the human brain using adaptive closed-loop stimulation. Prog. Neurobiol. 216, 102311. 10.1016/j.pneurobio.2022.102311 35750290

[B45] Nejati V. Salehinejad M. Nitsche M. Najian A. Javadi A. (2020). Transcranial direct current stimulation improves executive dysfunctions in adhd: Implications for inhibitory control, interference control, working memory, and cognitive flexibility. J. Atten. Disord. 24, 1928–1943. 10.1177/1087054717730611 28938852

[B46] Nozari E. Bertolero M. A. Stiso J. Caciagli L. Cornblath E. J. He X.. (2020). Is the brain macroscopically linear? a system identification of resting state dynamics. arXiv. [preprint]. 10.1101/2020.12.21.423856

[B47] Nunez P. Srinivasan R. (2006). Electric Fields of the Brain: The Neurophysics of EEG. New York – Oxford: Oxford University Press.

[B48] Paulus W. (2011). Transcranial electrical stimulation (tes – tdcs; trns, tacs) methods. Neuropsychol.Rehabilitat. 21, 602–617. 10.1080/09602011.2011.557292 21819181

[B49] Podilchak S. K. Frank B. M. Freundorfer A. P. Antar Y. M. (2009). “High speed metamaterial-inspired negative group delay circuits in cmos for delay equalization,” in 2009 2nd Microsystems and Nanoelectronics Research Conference. Ottawa, ON: IEEE. p. 9–12.

[B50] Popivanov D. Dushanova J. Mineva A. Krekule I. (1996). “Detection of successive changes in dynamics of eeg time series: linear and nonlinear approach,” in Proceedings of 18th Annual International Conference of the IEEE Engineering in Medicine and Biology Society. Amsterdam: IEEE. p. 1590–1591.

[B51] Potok W. van der Groen O. Bächinger M. Edwards D. Wenderoth N. (2022). Transcranial random noise stimulation modulates neural processing of sensory and motor circuits, from potential cellular mechanisms to behavior: a scoping review. eNeuro. 9, ENEURO.0248-21.2021. 10.1523/ENEURO.0248-21.2021 34921057 PMC8751854

[B52] Prosky J. Cagle J. Sellers K. Gilron R. de Hemptinne C. Schmitgen A.. (2021). Practical closed-loop strategies for deep brain stimulation: lessons from chronic pain. Front. Neurosci. 15, 762097. 10.3389/fnins.2021.762097 34975374 PMC8716790

[B53] Riedinger J. Hutt A. (2022). Mathematical model insights into eeg origin under transcranial direct current stimulation (tdcs) in the context of psychosis. J. Clin. Med. 11, 1845. 10.3390/jcm11071845 35407453 PMC8999473

[B54] Scangos K. Khambhati A. Daly P. Makhoul G. Sugrue L. Zamanian H.. (2021). Closed-loop neuromodulation in an individual with treatment-resistant depression. Nat. Med. 27, 1696–1700. 10.1038/s41591-021-01480-w 34608328 PMC11219029

[B55] Schier J. (1997). Estimation of transport delay using parallel recursive modified gram-schmidt algorithm. Int. J. Adapt. Control Signal Proc. 11, 431–442. 10.1002/(SICI)1099-1115(199708)11:5<431::AID-ACS417>3.0.CO;2-Q

[B56] Schulman J. J. Cancro R. Lowe S. Lu F. Walton K. D. Llinás R. R. (2011). Imaging of thalamocortical dysrhythmia in neuropsychiatry. Front. Hum. Neurosci. 5, 69. 10.3389/fnhum.2011.00069 21863138 PMC3149146

[B57] Smith O. J. (1959). A controller to overcome dead time. ISA J. 6, 28–33.

[B58] Sönmez S. Ayasun S. (2015). Stability region in the parameter space of pi controller for a single-area load frequency control system with time delay. IEEE Transact. Power Syst. 31, 829–830. 10.1109/TPWRS.2015.2412678

[B59] Stagg C. Antal A. Nitsche M. (2018). Physiology of transcranial direct current stimulation. J. ECT. 34, 144–152. 10.1097/YCT.0000000000000510 29877965

[B60] Stanslaski S. Farooqi H. Sanabria D. Netoff T. (2022). Fully closed loop test environment for adaptive implantable neural stimulators using computational models. J. Med. Device. 16, 034501. 10.1115/1.4054083 35646224 PMC9125865

[B61] Su F. Kumaravelu K. Wang J. Grill W. M. (2019). Model-based evaluation of closed-loop deep brain stimulation controller to adapt to dynamic changes in reference signal. Front. Neurosci. 13, 956. 10.3389/fnins.2019.00956 31551704 PMC6746932

[B62] Sun F. Morrell M. (2014). Closed-loop neurostimulation: the clinical experience. Neurotherapeutics 11, 553–563. 10.1007/s13311-014-0280-3 24850309 PMC4121459

[B63] Tervo A. E. Nieminen J. O. Lioumis P. Metsomaa J. Souza V. H. Sinisalo H.. (2022). Closed-loop optimization of transcranial magnetic stimulation with electroencephalography feedback. Brain Stimul. 15, 523–531. 10.1016/j.brs.2022.01.016 35337598 PMC8940636

[B64] Van Loan C. (1978). Computing integrals involving the matrix exponential. IEEE Trans. Automat. Contr. 23, 395–404. 10.1109/TAC.1978.1101743

[B65] Voss H. U. (2017). A universal negative group delay filter for the prediction of band-limited signals. arXiv. [preprint].30501209

[B66] Wagner T. Eden U. Rushmore J. Russo C. J. Dipietro L. Fregni F.. (2014). Impact of brain tissue filtering on neurostimulation fields: a modeling study. Neuroimage. 85, 1048–1057. 10.1016/j.neuroimage.2013.06.079 23850466 PMC4063680

[B67] Welch P. (1967). The use of fast fourier transform for the estimation of power spectra: A method based on time averaging over short, modified periodogram. Trans. Audio Electroacoustics. AU-15, 70–73. 10.1109/TAU.1967.1161901

[B68] Westover M. B. Kim S.-E. Ching S. Purdon P. L. Brown E. N. (2015). Robust control of burst suppression for medical coma. J. Neural Eng. 12, 046004. 10.1088/1741-2560/12/4/046004 26020243 PMC4517835

[B69] Yang Y. Connolly A. T. Shanechi M. M. (2018). A control-theoretic system identification framework and a real-time closed-loop clinical simulation testbed for electrical brain stimulation. J. Neural Eng. 15, 066007. 10.1088/1741-2552/aad1a8 30221624

[B70] Yang Y. Lee J. T. Guidera J. A. Vlasov K. Y. Pei J. Brown E. N.. (2019). Developing a personalized closed-loop controller of medically-induced coma in a rodent model. J. Neural Eng. 16, 036022. 10.1088/1741-2552/ab0ea4 30856619

[B71] Zhu Y. Wang J. Li H. Liu C. Grill W. M. (2021). Adaptive parameter modulation of deep brain stimulation based on improved supervisory algorithm. Front. Neurosci. 15, 750806. 10.3389/fnins.2021.750806 34602976 PMC8481598

