# Supplementary material for: Delayed closed-loop neurostimulation for the treatment of pathological brain rhythms in mental disorders: a computational study
Source: Front Neurosci. 2023 Jul 5;17:1183670. doi: 10.3389/fnins.2023.1183670 (PMC10354341; doi:10.3389/fnins.2023.1183670)
Supplement: Supplementary file 1 [file Data_Sheet_1.pdf]

Supplementary Material

CORTICO-THALAMIC BRAIN MODEL DETAILS

The differential equation system (13) develops as

$$\begin{aligned}
\tau_e \frac{dV_e(t)}{dt} &= -V_e(t) + F_e T_c(V_e(t) - V_i(t)) + F_{ct} T_{th}(V_{th,e}(t) - V_{th,i}(t)) + F_{ccx} S_e(v(t)) + \mu_e + I_e + \xi_e(t) + b_1 u(t) \\
\tau_i \frac{dV_i(t)}{dt} &= -V_i(t) + F_i T_c(V_e(t) - V_i(t)) + \mu_i + I_i + \xi_i(t) + b_2 u(t) \\
\tau_{th,e} \frac{dV_{th,e}(t)}{dt} &= -V_{th,e}(t) + F_{tc} T_c(V_e(t-\tau) - V_i(t-\tau)) + \mu_{th,e} + \xi_{th,e}(t) \\
\tau_{th,i} \frac{dV_{th,i}(t)}{dt} &= -V_{th,i}(t) + F_{tr} T_{ret}(V_{ret}(t)) + \mu_{th,i} + \xi_{th,i}(t) \\
\tau_{ret} \frac{dV_{ret}(t)}{dt} &= -V_{ret}(t) + F_{rt} T_{th}(V_{th,e}(t) - V_{th,i}(t)) + F_{rc} T_c(V_e(t-\tau) - V_i(t-\tau)) + \mu_{ret} + \xi_{ret}(t) \\
\tau_{ce} \frac{dv(t)}{dt} &= -v(t) + F_{cx} S_e(v(t)) - M_{cx} S_i(w(t)) + M_{cx,th} T_{th}(V_{th,e}(t-\tau) - V_{th,i}(t-\tau)) + \mu_{ce} + I_{ce} + \xi_{ce}(t) + b_3 u(t) \\
\tau_{ci} \frac{dw(t)}{dt} &= -w(t) - F_{cx} S_i(w(t)) + M_{cx} S_e(v(t)) + \mu_{ci} + I_{ci} + \xi_{ci}(t) + b_4 u(t)
\end{aligned} \tag{S1}$$

where the transfer functions are defined as

$$\begin{aligned}
T_m(x) &= \frac{1}{2}\left(1 - \mathrm{erf}\left(-\frac{x}{\sqrt{2}\sigma_m}\right)\right) \\
S_m(x) &= \frac{1}{2}\left(1 - \mathrm{erf}\left(-\frac{x}{\sqrt{2}\sigma_{cm}}\right)\right).
\end{aligned} \tag{S2}$$

The ξ_x terms represent the driving noises, which are uncorrelated Gaussian noise defined as

$$\langle \xi_x(t) \rangle = 0 \quad , \quad \langle \xi_x(t)\xi_y(t') \rangle = \frac{Q_x}{N}\delta_{xy}\delta(t-t'),$$

with $x = e, i, (th, e), (th, i), ret, ce, ci$. The variances in Eq. (S2) are defined as

$$\sigma_c^2 = \frac{Q_e}{\tau_e} + \frac{Q_i}{\tau_i}, \qquad \sigma_{th}^2 = \frac{Q_{th,e}}{\tau_{th,e}} + \frac{Q_{th,i}}{\tau_{th,i}}, \qquad \sigma_{ret}^2 = \frac{Q_{ret}}{\tau_{ret}}$$

$$\sigma_{ce}^2 = \frac{Q_{ce}}{\tau_{ce}}, \qquad \sigma_{ci}^2 = \frac{Q_{ci}}{\tau_{ci}}$$

All the parameters are given in Table S1.

parameter	description	value
τ_e	exc. decay time (infragranular)	10 ms
τ_i	inh. decay time (infragranular)	50 ms
$\tau_{th,e}$	exc. decay time (relay)	5 ms
$\tau_{th,i}$	inh. decay time (relay)	30 ms
τ_{ret}	exc. decay time (reticular)	8 ms
τ_{ce}	exc. decay time (supragranular)	5 ms
τ_{ci}	inh. decay time (supragranular)	20 ms
τ	cortico-thalamic propagation delay	40 ms
F_e	exc. synaptic strength	1.0
F_i	inh. synaptic strength	2.0
F_{ct}	synaptic strength (relay $\rightarrow$ cortex)	1.2
F_{tc}	synaptic strength (cortex $\rightarrow$ relay))	1.0
F_{tr}	synaptic strength (reticular $\rightarrow$ relay)	1.0
F_{rt}	synaptic strength (relay $\rightarrow$ reticular)	0.3
F_{rc}	synaptic strength (cortex $\rightarrow$ reticular)	0.6
F_{cx}	synaptic strength (exc. $\rightarrow$ exc.)	2.18
M_{cx}	synaptic strength (inh. $\rightarrow$ exc.)	3.88
F_{ccx}	synaptic strength (supragranular $\rightarrow$ infragranular)	0.05
$F_{cx,th}$	synaptic strength (thalamic relay $\rightarrow$ supragranular)	0.1
μ_e	exc. noise input (infragranular)	0.1
μ_i	inh. noise input (infragranular)	0.0
$\mu_{th,e}$	exc. noise input (relay)	1.3
$\mu_{th,i}$	inh. noise input (relay)	1.0
μ_{ret}	exc. noise input (reticular)	0.0
μ_{ce}	exc. noise input (supragranular)	0.05
μ_{ci}	inh. noise input (supragranular)	0.05
I_e	exc. resting input (infragranular)	2.7
I_i	inh. resting input (infragranular)	1.7
I_{ce}	exc. resting input (supragranular)	1.1
I_{ci}	inh. resting input (supragranular)	0.4
Q_e	exc. input noise variance (infragranular) (pathological)	3×10^{-5}
Q'_e	exc. input noise variance (infragranular) (healthy)	5×10^{-5}
Q_i	inh. input noise variance (infragranular)	0.001
$Q_{th,e}$	exc. input noise variance (relay) (pathological)	2.5×10^{-6}
$Q'_{th,e}$	exc. input noise variance (relay) (healthy)	1.2×10^{-5}
$Q_{th,i}$	inh. input noise variance (relay)	12.6×10^{-6}
Q_{ret}	exc. input noise variance (reticular)	10.9×10^{-6}
Q_{ce}	exc. input noise (supragranular)	2×10^{-5}
Q_{ci}	inh. input noise (supragranular) (pathological)	8×10^{-5}
Q'_{ci}	inh. input noise (supragranular) (healthy)	1×10^{-6}
N	number of neurons	1000
$b_{1,2,3,4}$	input coupling constants	1
c_1	observation coefficient (supragranular)	0.3
c_3	observation coefficient (infragranular)	1

Table S1. Parameter set of model (S1). The choice of parameters is for the most part based on the paper in which it was developed Riedinger and Hutt (2022)

REFERENCES

Riedinger, J. and Hutt, A. (2022). Mathematical model insights into eeg origin under transcranial direct current stimulation (tdcs) in the context of psychosis. *Journal of Clinical Medicine* 11, 1845. doi:http://dx.doi.org/10.3390/jcm11071845
